# Fully organic compliant dry electrodes self-adhesive to skin for long-term motion-robust epidermal biopotential monitoring

**DOI:** 10.1038/s41467-020-18503-8

**Published:** 2020-09-17

**Authors:** Lei Zhang, Kirthika Senthil Kumar, Hao He, Catherine Jiayi Cai, Xu He, Huxin Gao, Shizhong Yue, Changsheng Li, Raymond Chee-Seong Seet, Hongliang Ren, Jianyong Ouyang

**Affiliations:** 1 Department of Materials Science & Engineering, National University of Singapore, Faculty of gineering, 7 Engineering Drive 1, Singapore, 117574 Singapore; 2 Department of Biomedical Engineering, National University of Singapore, Faculty of Engineering, 4 Engineering Drive 3, Singapore, 117583 Singapore; 3 Singapore Institute of Manufacturing Technology, A*STAR Singapore, Fusionopolis Two, 4 Fusionopolis Way, Singapore, 138635 Singapore; 4 National University of Singapore (Suzhou) Research Institute (NUSRI), Suzhou, China; 5 The Chinese University of Hong Kong (CUHK) Robotics Institute, Shatin, Hong Kong; 6 Beijing Advanced Innovation Center for Intelligent Robots and Systems & School of Mechatronical Engineering, Beijing Institute of Technology, Beijing, 100081 China; 7 Division of Neurology, Department of Medicine, National University Health System, Singapore, Singapore; 8 Department of Medicine, Yong Loo Lin School of Medicine, National University of Singapore, Singapore, Singapore

**Keywords:** Electrocardiography - EKG, Electroencephalography - EEG, Electromyography - EMG, Electronic devices

## Abstract

Wearable dry electrodes are needed for long-term biopotential recordings but are limited by their imperfect compliance with the skin, especially during body movements and sweat secretions, resulting in high interfacial impedance and motion artifacts. Herein, we report an intrinsically conductive polymer dry electrode with excellent self-adhesiveness, stretchability, and conductivity. It shows much lower skin-contact impedance and noise in static and dynamic measurement than the current dry electrodes and standard gel electrodes, enabling to acquire high-quality electrocardiogram (ECG), electromyogram (EMG) and electroencephalogram (EEG) signals in various conditions such as dry and wet skin and during body movement. Hence, this dry electrode can be used for long-term healthcare monitoring in complex daily conditions. We further investigated the capabilities of this electrode in a clinical setting and realized its ability to detect the arrhythmia features of atrial fibrillation accurately, and quantify muscle activity during deep tendon reflex testing and contraction against resistance.

## Abstract (web summary)

Reported wearable dry electrodes have limited long-term use due to their imperfect skin compliance and high motion artifacts. Here, the authors report an intrinsically conductive, stretchable polymer dry electrode with excellent self-adhesiveness for long-term high-quality biopotential detection.

## Introduction

Human biopotentials such as electrocardiography (ECG)^1^, electromyography (EMG)^2^, and electroencephalography (EEG)^3^ are significant for diagnosis and treatment of heart-, brain-, and muscle-related diseases^4^. These biopotentials can be transduced by electrically interfacing with the skin via epidermal electrodes. An efficient wearable electrode is crucial for accurately recording these biopotential signals, especially in the case of continuous monitoring of inconspicuous heart diseases and rehabilitation in daily life. At present, Ag/AgCl gel electrodes are predominant in a clinical setting to obtain surface biopotentials, but prone to signal degradation in the long run of continuous monitoring due to the volatilization of the liquid in gel electrolyte and skin irritation^5^. Hence, significant effort has been devoted to the development of skin-friendly dry electrodes for biopotential measurements^6,7^. The dry electrodes in literature can be classified mainly into dry contact electrodes and dry capacitive (noncontact) electrodes^8^. The dry capacitive electrodes give rise to motion artifacts quite sensitive to body movement, and thus are not suitable for biopotential monitoring. The dry contact electrodes mainly include thin metal films, conductive polymers composites, and intrinsically conductive polymers. Although thin metal films can have high conductivity, they are not stretchable and adhesive^9^. As a result, high noise can be observed on the biopotential signals, particularly during body movement.

The research works on dry contact electrodes have been focused on soft conductive polymer composites and intrinsically conductive polymers due to their adaptation to rough and even deformed skin^9,10^. The conductive polymer composites consist of elastomers and conductive nanofillers like metals^11^, nanotubes^12^, nanowires^13^, and nanosheets^14^. The conductive nanofillers are the minority in the elastomer matrix, leading to a small effective contact area between the conductive nanofillers and human skin. As a result, the electrode–skin interface impedance is higher than that with Ag/AgCl gel electrode by a couple of orders in magnitude, and significant effect can be observed on the biopotential signals^15^. Mismatching between a dry electrode and human skin can occur during body movement, which can be improved if the dry electrodes are adhesive to human skin. Polymer composite patches with bio-inspired micro-pillar or sucker-like structures can be stretchable and adhesive^16,17^. Nevertheless, their adhesion to the skin is easily affected by secreted sweat or dirt on the skin, and clustering or contamination of the structures^18^. In addition, the suction induced adhesion of these structures can cause discomfort to patients. There is also concern about the toxicity of the nanofillers^19,20^.

Intrinsically conductive polymers can have high effective contact areas with human skin, biocompatibility, high electrical conductivity, and inherent mechanical flexibility^21–23^. Among them, poly(ethylenedioxythiophene):poly(styrenesulfonate) (PEDOT:PSS) has gained particular attention as dry electrodes. For example, PEDOT:PSS films printed on paper or polyimide foil were studied as the ECG dry electrodes^2,24^. However, the signal has poor quality and the electrodes may delaminate from the skin, because the PEDOT:PSS films are not adhesive and stretchable. Greco et al. reported that ultrathin films of ethyl cellulose/PEDOT:PSS bilayer could be adhesive to skin and be used as the EMG dry electrode^25^. However, EMG signal is susceptible to strain during muscle movement because of the limited stretchability of ethyl cellulose/PEDOT:PSS bilayer. In addition, it is very difficult to handle ultrathin films. To obtain high-quality biopotential signals, a dry electrode should be conductive, biocompatible, stretchable, conformable, and self-adhesive to the skin. Nevertheless, intrinsically conductive polymers are neither stretchable nor adhesive to the skin.

In this work, we fabricated a fully-organic, self-adhesive, and stretchable dry electrode with high conductivity by solution processing of biocompatible blends of PEDOT:PSS, waterborne polyurethane (WPU) and D-sorbitol. It possesses high conductivity and skin-compliant stretchability, with appreciated adhesion on dry and wet skin conditions, respectively. Therefore, the prepared dry electrode shows lower contact impedance on the skin and much lower noise level in static and dynamic detection than other dry electrodes in literature and standard Ag/AgCl gel electrodes. This dry electrode can always give rise to high-quality epidermal biopotential signals, including ECG, EMG, and EEG, in various conditions such as dry and wet skin and during body movement. Moreover, this dry electrode can precisely identify the arrhythmia of a patient with atrial fibrillation and muscle activity in a clinical setting.

## Results

### Fabrication and characterization

**Fig. 1:**
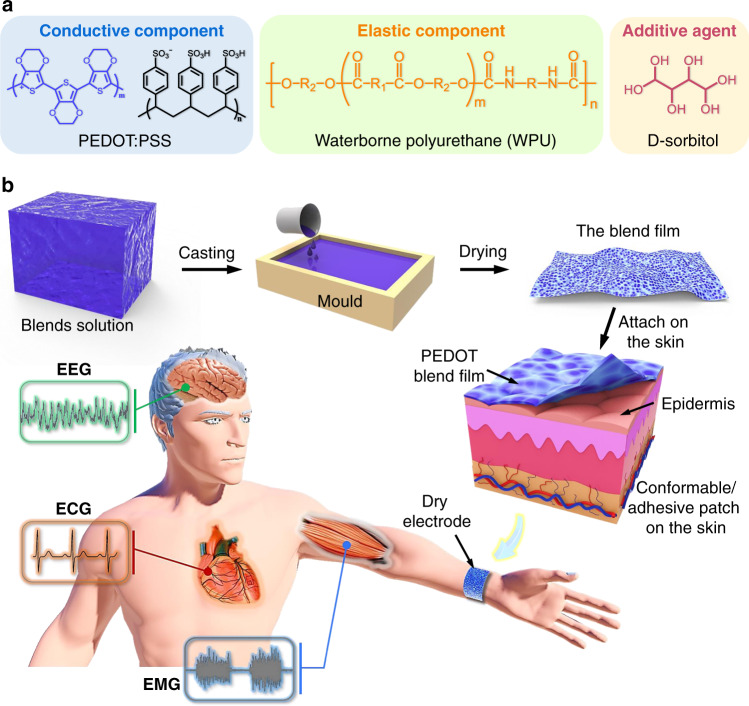
Schematic illustration for the preparation of PEDOT:PSS/WPU/D-sorbitol films. **a** Chemical structures of PEDOT:PSS, WPU, and D-sorbitol. **b** Fabrication of the PWS blend films: Firstly, mixing of PEDOT:PSS, WPU and D-sorbitol; Secondly, drop-casting the blend solution in a mold; Thirdly, drying at 60 °C. The resulting blend film can be used as an adhesive electrode on the skin for epidermal biopotential detections such as electrocardiography (ECG), electromyography (EMG), and electroencephalography (EEG).

The fabrication process of the self-adhesive dry electrode is illustrated in Fig. 1. Although PEDOT:PSS is intrinsically conductive, it has very limited stretchability and is not adhesive^26^. Nonionic WPU can mix well with PEDOT:PSS solution (Supplementary Fig. S1) and improve the stretchability of the PEDOT:PSS film^27^. D-sorbitol is further blended into the mixture to further increase its stretchability^28^. Moreover, D-sorbitol can enhance the adhesiveness of the polymer film on the substrates. Uniform blend films can be prepared by casting aqueous solution consisting of PEDOT:PSS, WPU, and D-sorbitol (Fig. 1a, b). PWS is used to represent the blend of PEDOT:PSS, WPU, and D-sorbitol. The PWS blend films are then investigated as adhesive and stretchable dry electrodes for epidermal biopotentials, including ECG, EMG, and EEG (Fig. 1b).

**Fig. 2:**
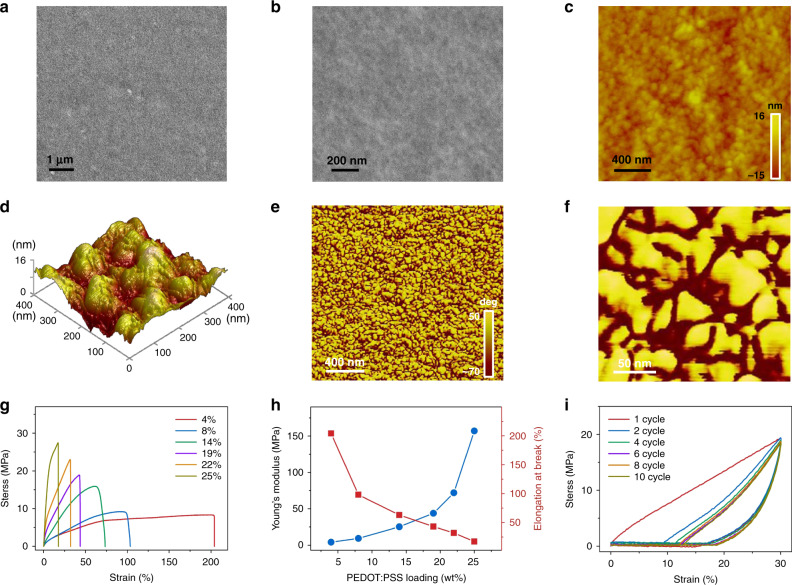
Characterization and mechanical properties of PWS films. **a**, **b** SEM image of a PWS film. **c** Topology AFM images of a PWS film. **d** 3D topographical AFM image. **e**, **f** Phase AFM images of a PWS film. **g** Stress-strain curves of PWS films with different PEDOT:PSS loadings. **h** Young’s modulus and elongation at break of PWS films with respect to the PEDOT:PSS loading. **i** Tensile stress–strain curves of PWS films in the first 10 cycles. The tensile speed was 50 mm/min. The PEDOT:PSS loading was 19 wt% for **a**–**f**, and **i**.

The PWS films were characterized by scanning electron microscopy (SEM) and atomic force microscopy (AFM). The SEM image indicates nanoscale grainy morphology (Fig. 2a, b). The grains have a size of ~100 nm in terms of the topological AFM image (Fig. 2c). The surface roughness is about 16 nm (Fig. 2d). Remarkably, the phase AFM image reveals the presence of two phases in the blend (Fig. 2e, f)^29^, because PEDOT:PSS and WPU form a colloidal structure in aqueous solution (Supplementary Fig. S1)^30^. This phase structures are supported by the dependence of the phase volume proportion on the loading of PEDOT:PSS in the blends. Higher PEDOT:PSS loading gives rise to a more dark-colored phase (Supplementary Fig. S2). Thus, the dark-colored phase is dominant with PEDOT:PSS, while the light-color phase is mainly due to WPU. The PEDOT chains form conductive networks in the blend film. The presence of the two continuous phases in the blend film is further supported by the element distribution of nitrogen of WPU and sulfur of PEDOT:PSS as revealed by the energy-dispersive X-ray (EDX) results (Supplementary Fig. S3). Similar microstructure and element distribution were observed by the cross-section SEM images and EDX as well (Supplementary Fig. S4).

Mechanical and electrical properties The stress–strain curves of PWS films and PEDOT:PSS/WPU (PW) films without D-sorbitol are shown in Fig. 2g and Supplementary Fig. S5a, respectively. With the increase of the PEDOT:PSS loading, the elongation at break decreases while the Young’s modulus increases for both PWS and PW films. The elongation at break is about 28% for the PW films with 30 wt% of PEDOT:PSS (Supplementary Fig. S5b). The addition of 38 wt% D-sorbitol into this blend can increase the elongation at break to 43% (Fig. 2h). This stretchability is on par with the stretchability of the human skin (~30%)^31^. However, a further increase in D-sorbitol loading can cause remarkable moisture absorption, and make the PWS films volatile and hence susceptible to breaking. Therefore, the optimal loading of D-sorbitol in the blend is 38 wt%. The PWS films with the optimal WPU and D-sorbitol loadings can be stretched repeatedly. As shown in Fig. 2i, although hysteresis can be observed in the first stress-strain cycle, the stretchable behavior becomes stable in the subsequent cycles.

**Fig. 3:**
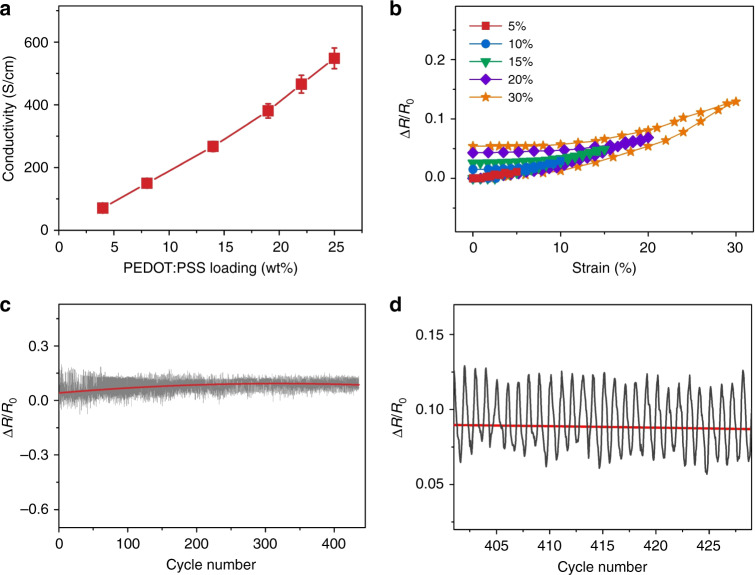
Electrical properties of PWS films. **a** Dependence of the conductivity of the PWS films on the PEDOT:PSS loading. **b** Variations of the resistance of PWS films with the strains. The PWS films were stretched to different maximum strains of 5, 10, 15, 20, and 30% in different cycles. Resistance variation of a PWS film in **c** repeated stretching/releasing cycles and **d** in the 400th–430th stretching/releasing cycles. The PWS film was stretched to the strain of 30% in each cycle, and the tensile speed was 50 mm/min. The PEDOT:PSS loading was 19 wt% for **b**, **c**, and **d**.

The conductivity of the PWS films depends on the PEDOT:PSS loading as well. The conductivity increases almost linearly from 72 to 545 S/cm when the PEDOT:PSS loading is increased from 4 to 25 wt% (Fig. 3a). This is consistent with the continuous phase structure of PEDOT:PSS in the PWS films. If PEDOT:PSS is instead dispersed as a minority phase in the matrix of WPU, the conductivity of the PWS film should drastically increase until the PEDOT:PSS loading reaches the percolation threshold^32^. Since the skin deformation for human motion in daily life is usually less than 30%, the PWS films with the PEDOT:PSS loading of 19 wt% are investigated for the application as a dry electrode. In the strain range of 30%, the resistance variation is less than 5.5% (Fig. 3b, Supplementary Movie 1). The resistance in the first three cycles remains almost the same. In the repeated stretching and releasing cycles, the PWS electrodes exhibit stable conductivity (Supplementary Movie 2). After 440 stretching/releasing cycles, the variation of the conductance is ~6.5% along the horizontal direction of the PWS film (Fig. 3c, d). The conductance variation along the vertical direction can be even smaller^33^.

The PEDOT networks in the PWS films do not remarkably change in the tensile study. The morphology of a PWS film was studied by SEM and phase AFM before and after stretching to a strain of 30% (Supplementary Fig. S6). No remarkable change can be observed by SEM (Supplementary Fig. S6a, b). The phase AFM images indicate the continuous PEDOT networks in the relaxed or stretched PWS film (Supplementary Fig. S6c–f). This small resistance variation with strain is similar to the conductive PEDOT organogels that have continuous PEDOT networks inside^34^.

### Adhesive properties

**Fig. 4:**
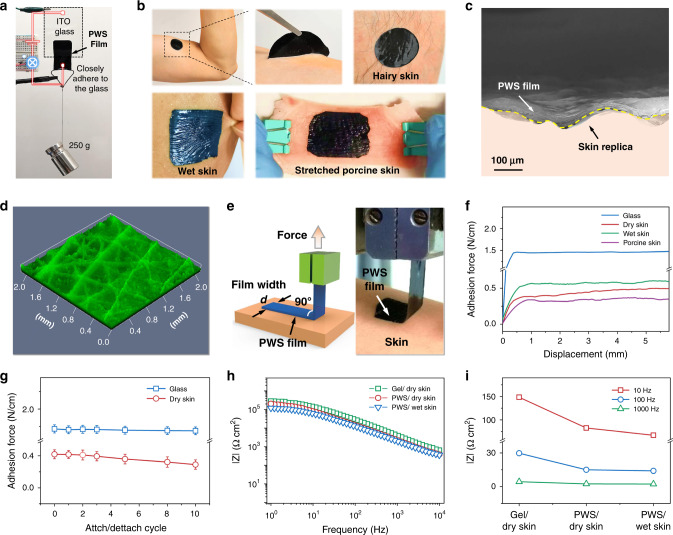
Conformability and adhesiveness of PWS films. **a** A PWS film bearing 250 g of weight attach tightly on the ITO glass, enabling the LED light in the conducting circuit. **b** The adhesiveness of a PWS film on various skins including the smooth skin, hairy rough skin, wet deformable skin, and stretched porcine skin. **c** Cross-section SEM image of a PWS film conformed on a rough skin replica. **d** 3D optical image of a PWS film replicated the skin wrinkles. **e** The setup for the measurement of the interfacial adhesion on the skin or glass by the standard 90-degree peel test (ASTM D2861). **f** Interfacial adhesion forces of PWS films on glass and various skins. **g** Adhesion forces of PWS films on glass and dry skin within ten repetitions of attaching/detaching. **h**, **i** Impedance spectra of commercial Ag/AgCl gel electrode and PWS dry electrode on dry and wet skin, and the corresponding impedances at 10, 100, and 1000 Hz.

The PWS films exhibit excellent adhesiveness on a glass substrate and skin. A PWS film of 2.5 × 2.5 cm^2^ and 22 ± 1 μm thick attached on an indium tin oxide (ITO) glass is used in an electrical circuit (Fig. 4a and Supplementary Movie 3). Even bearing an object of 250 g, the PWS film can attach tightly to the ITO glass and enable the LED in the circuit to work. Moreover, the PWS films can attach tightly on both smooth and hairy skin (Fig. 4b, Supplementary Movie 4). On the dry and wet skins with substantial wrinkles, the PWS films can adapt to the grooves of the wrinkles and adhere firmly (Supplementary Fig. S7, Fig. 4b). Even on a piece of stretched porcine skin (uniaxial tensile > 40%) simulating largely deformed human skin, the PWS film can adapt to the skin deformation and does not delaminate (Fig. 4b). The strain hardly affects the adhesion of the PWS films to the skin (Supplementary Fig. S8). The adhesion force of a pristine PWS film to the skin is 0.43 N/cm. It increases slightly to 0.46 N/cm when the PWS film is stretched to a strain of 30%. The increase in the adhesion force can be attributed to the change in film thickness by strain. The thickness of the pristine PWS film is 20 μm, and it decreases to 15 μm at a strain of 30%. The enhancement in the adhesion force of the stretched PWS film is attributed presumably to the better compliance of thinner film to skin^35^. Even after repeated stretching/releasing cycles, the PWS films still have stable adhesion on glass and skin. They can tightly attach to a wrist that bends and twists vigorously and continuously (Supplementary Movie 5, Movie 6).

To evaluate the contact of PWS film to skin microscopically, a silicon rubber was used as the skin replica. After placed on the skin replica and pressed for about 3 s, the PWS film could adapt well to the skin replica and exhibited similar skin wrinkle morphology. The cross-section SEM image indicates that the PWS film is conformable to the uneven and curved surface of the skin replica in the sub-millimeter scale (Fig. 4c). The 3D optical image taken with a confocal microscope displays clearly shows the replicated skin wrinkles on the PWS film detached from the skin replica (Fig. 4d).

The adhesiveness of the PWS films arises presumably from WPU and D-sorbitol because pristine PEDOT:PSS films are not adhesive. The adhesion mechanism can be attributed to the physical adsorption of PWS to the skin and the mechanical force between them. The surface composition of PWS film is characterized by the IR reflectance spectroscopy (Supplementary Fig. S9). The strong absorption band at around 3300 cm^−1^ is attributed mainly to the stretching vibration of O–H and N–H group of D-sorbitol and WPU, while the bands at 1725 and 1528 cm^−1^ can be assigned to the stretching vibration of C=O and the bending vibration of N–H of WPU, respectively^36^. Organic molecules and polymers with O–H, N–H, and C=O groups like cellulose adhesives^37^ and polyurethane adhesives^38^ can be adhesive due to the strong physical force to substrate. The PWS films with rich O–H, N–H, C=O groups on the surface can have strong physical adsorption to the outermost stratum corneum mostly consisted of keratin and lipids^39^. In addition, the soft PWS films can adapt well with the crevices of the skin, which not only increases the contact area between PWS and skin but also induces adhesive force between them.

The adhesion forces of PWS films to the skin is insensitive to the thickness as the film thickness above 20 μm (Supplementary Fig. S10). The adhesive forces of the PWS films on dry/wet skin and glass are evaluated by the interfacial adhesive force with the standard 90-degree peel testing method (ASTM D2861) (Fig. 4e). The adhesive force (*f*), *f* = F(peel force)/*d*(film width), is plotted against the displacement (*L*) (Fig. 4f). The PEDOT/WPU (PW) film without D-sorbitol has adhesive forces of 0.12 and 0.18 N/cm on skin and glass, respectively (Supplementary Fig. S11a, c). At the loading of 38 wt% D-sorbitol, the maximum adhesive forces of the PWS films approach 0.41 and 1.44 N/cm on skin and glass, respectively. The further increase of D-sorbitol loading in the PWS film decreases its adhesiveness. The adhesive force is 0.2 N/m on the skin at the D-sorbitol loading of 55 wt%. This force is attributed to the wet and slippery surface of the PWS film caused by the moisture absorption of excess D-sorbitol. The optimal D-sorbitol loading is 38 wt% in terms of the adhesive force (Supplementary Fig. S11b, d). The PWS films are adhesive even to wet skin. A wet skin is obtained by spraying water onto a volunteer’s forearm and then removing the large water droplets. The PWS film can have an adhesive force of 0.56 N/cm on this wet skin (Fig. 4f). After ten cycles of attaching/detaching, the adhesion of a PWS film on glass substrate hardly decreases and the adhesion on dry skin only decreases slightly (Fig. 4g). The adhesion reduction on the skin is mainly due to the dirt like sebum. After the contamination is removed by wiping the skin and the PWS electrode with a clinical-grade isopropyl alcohol swab, the adhesion is restored (Supplementary Fig. S12). Hence, the PWS films can be used as a dry electrode repeatedly.

The PWS films have low electrode-skin electrical impedances in the frequency range of 1–10^4^ Hz. Two circular PWS films with a diameter of 3 cm were placed on a volunteer’s forearm and their separation was 10 cm^40^. PWS films with the thicknesses of 12, 27, and 55 μm show that the impedances slightly decrease with decreasing film thickness (Supplementary Fig. S13). This can be attributed to the high conductivity of the PWS films, which is higher than the commercial Ag/AgCl gel electrode by several orders by magnitude. The PWS electrodes exhibits a lower impedance than that of the Ag/AgCl gel electrode (Fig. 4h, i). Their impedances at 10 Hz are 82 KΩ cm^2^ and 148 KΩ cm^2^, respectively. The impedance of the PWS films on skin is much lower than the stretchable dry electrodes in literatures^2,13^ (Supplementary Table S1). Compared with highly conductive nanocomposite electrodes with metal nanoparticles or nanowires, the PWS electrodes show significantly lower skin-contact impedance, although the conductivity of the latter can be lower than the former^41,42^. This is because the impedance is mainly related to the electrode–skin contact instead of the conductivity of the electrode material. The effective contact area between the conductive nanofillers of nanocomposites and skin is very small because the nanofillers are the minority with loading of usually <2 vol%^43^. The loading of the nanofillers cannot be too high, because more nanofillers will lower the stretchability/softness and the adhesiveness of the nanocomposites. Those dry electrodes in literature do not have the other merits of the PWS films, such as the mechanical stretchability and the self-adhesiveness. In addition, the impedance of the PWS films on skin hardly changes over a long period (Supplementary Fig. S14). The impedance slightly decreases in the first 10 min after a PWS film is attached to a skin, which mainly arises from the secretion of sweat on the skin^44^. The impedance is then quite stable over time. Therefore, the PWS films can be used as dry electrodes for long-term healthcare monitoring.

### Biopotential detection using PWS dry electrodes

**Fig. 5:**
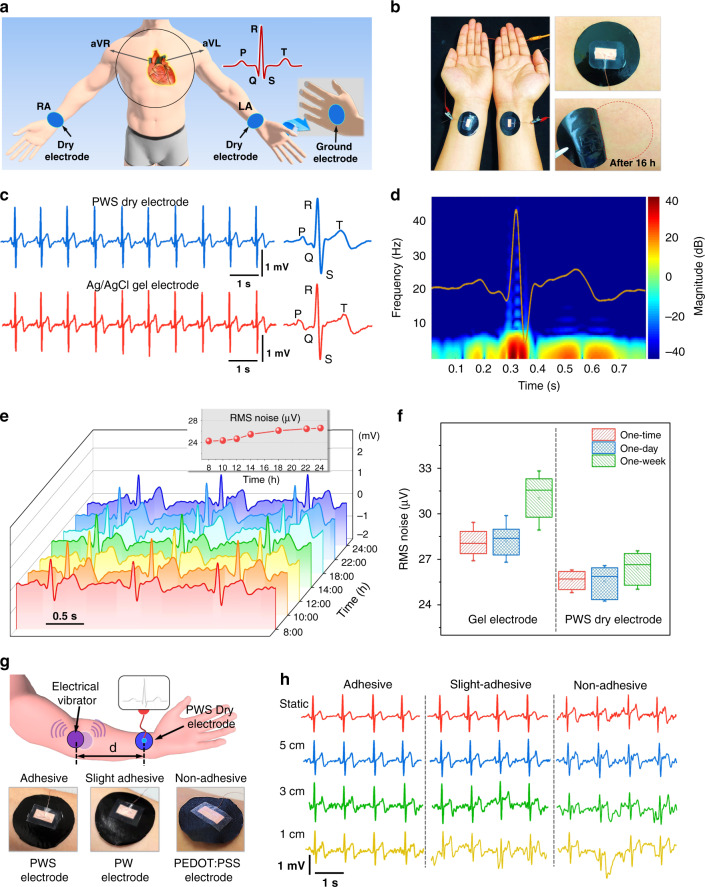
ECG detection using PWS dry electrodes. **a** Schematic illustration of the ECG detection. **b** Photos of PWS dry electrodes. They could attach firmly to the skin of a wrist and then peeled off after 16 h. No skin irritation or visible redness was observed after the use of 16 h. **c** Comparison of ECG signals using a PWS dry electrode and commercial Ag/AgCl gel electrode. **d** Spectrogram of the ECG pulse recorded using the PWS dry electrode. **e** Long-term monitoring of ECG using PWS dry electrodes for 1 day and their RMS noise. **f** The RMS noise picked by Ag/AgCl gel electrode and PWS dry electrode during ECG recording in one-time, 1-day, and 1 week. **g**, **h** ECG testing on the skin under motion induced by an electrical vibrator. The distance of the vibrator from the electrode was 5, 3, or 1 cm.

The PWS films can be used as wearable dry electrodes to detect epidermal biopotentials. To record ECG signals, two circular PWS films with a diameter of 3 cm were placed symmetrically on a volunteer’s inner wrists of the right and left arms, and another PWS film was attached on the back of the left hand as the ground electrode (Fig. 5a, b). Owing to the biocompatibility and conformability^45–47^, the PWS electrodes hardly irritate the skin, and no redness is observed even after prolonged use of 16 h (Fig. 5b). The PWS electrodes The PWS electrodes give rise to high-quality ECG signals with the PQRST waveforms and a peak-to-peak voltage (QRS complex) of 1.84 mV (Fig. 5c). These ECG waveforms are nearly the same as that using the standard Ag/AgCl gel electrodes. In addition, the spectrogram of the ECG pulse in the frequency range of 0–45 Hz is obtained by Fourier transformation (Fig. 5d). The clear frequency identification of PQRST peaks is distinguishable along with the power of the signal in 20–40 dB, and these are critical in clinical settings to diagnose various cardiac signal abnormalities such as congenital heart defects, cardiac arrhythmia, or potential heart failure.

The PWS electrodes can be used for long-term healthcare monitoring, as supported by the high-quality ECG signals after continuous use for 16 h (Fig. 5e) and throughout at least 1-month (Supplementary Fig. S15).

The noise of the ECG signal can be evaluated by the root-mean-squared (RMS) analysis, which indicates the fluctuations of the signal over time. The RMS noise picked using the PWS electrodes is about 25 μV, which is even lower than that of Ag/AgCl gel electrodes (28 μV) (Fig. 5f). It is also much lower than that of other dry electrodes in the literature (Supplementary Table S1). This noise only increases to 27 μV after 1 week (Fig. 5f), while it increases to 32 μV for the Ag/AgCl electrodes. Therefore, the PWS electrodes are much better than the Ag/AgCl electrodes for long term monitoring^48–51^. The signal quality is also much better than the present dry electrodes using PEDOT or nanocomposites (Supplementary Table S1)^2,13,16,17,24,52–57^.

ECG signals were detected during body movement. The body movement was induced by firmly attaching a disc-shaped electromechanical vibrator on the skin (Fig. 5g). The vibrator induced a mean swing amplitude of about 1.5 mm to the skin. The vibration of the skin under the PWS electrode depends on its distance from the vibrator. When the distance (*d*) is smaller, the skin vibration is more vigorous. ECG signals were recorded at the distance of 5, 3, and 1 cm, respectively (Fig. 5h), and the corresponding noise levels are shown in Supplementary Fig. S16. The PQRST waveforms are distinguishable without remarkable drift in the baseline, even at the shortest distance of 1 cm. The RMS noise obtained from PWS dry electrodes is below 38 μV, showing high resistance against motion artifacts interferences, which is much better than other dry electrodes (Supplementary Table S1). The motion artifacts are related to the adhesiveness of the dry electrodes. When slightly adhesive PEDOT:PSS/WPU (PW) films or nonadhesive PEDOT:PSS films are used as the electrodes, significant motion artifacts appear (Fig. 5h). The baseline fluctuations and the noise are even worse when the vibrator is further closer to the electrode. When a PWS film is attached to the skin, it is stretched during the skin movement, such as driven by wrist bending or twisting, which only slightly affects the resistance and adhesion of the PWS electrode (Supplementary Fig. S17a). The possible hysteresis in the stress-strain behavior of the PWS film due to the repeated stressing/releasing cycle has little influence on the contact impedance and does not increase the motion artifacts (Supplementary Fig. S17b).

The PWS electrodes were further placed on wet skin for ECG testing (Supplementary Fig. S18), as the accurate measurement on the wet and sweaty skin is also a concern for long-term healthcare monitoring. A volunteer’s forearm was sprayed with water, and the excess water droplets were removed, leaving a wet skin. The ECG signal on wet skin is almost the same as on dry skin. The ECG signal is not affected when the wrist bends at an angle of 30°, 60°, and 90°. ECG signals can be recorded even when the PWS electrodes attached to the wrist and opisthenar were immersed in water (Supplementary Fig. S19a). The PQRST waveforms and stable baseline are observable, with the signal quality saliently higher than that with the commercial Ag/AgCl gel electrodes (Supplementary Fig. S19b).

**Fig. 6:**
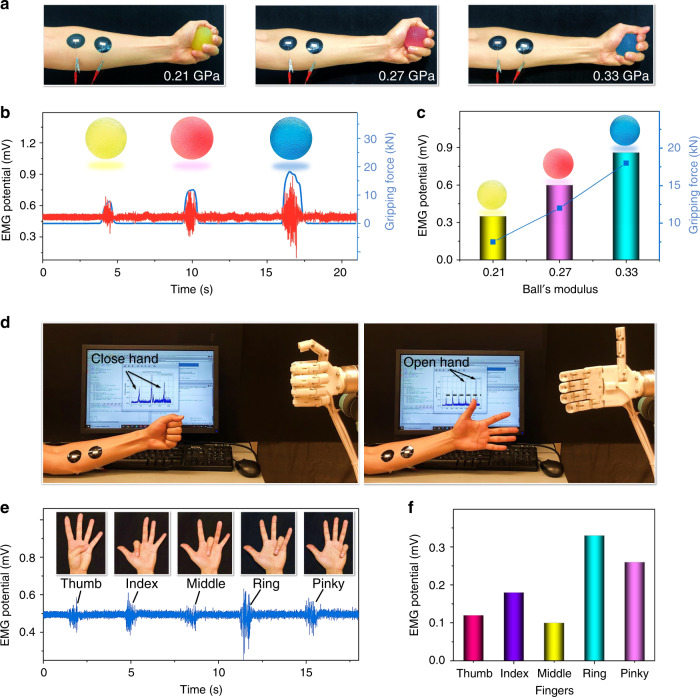
EMG measurements using PWS dry electrodes. **a** Monitoring of the EMG signal on a forearm gripping a ball. The three balls had different moduli of 0.21, 0.27, and 0.33 GPa, respectively. **b** EMG signals while gripping the balls. **c** Variations of the EMG signal amplitude and the gripping force with the modulus of the balls. **d** Using EMG signals to control the motion of a robotic hand, including opening and closing. **e** EMG signals produced by the flexion/extension of different fingers. **f** EMG signal intensities produced by the five fingers.

The PWS films can further be used as dry electrodes for EMG that detects the action potential generated by the muscle fibers. As shown in Fig. 6a, two PWS electrodes were placed on the wrist flexors muscles (inner side of the forearm) of a volunteer. When the hand gripes a ball, the wrist flexors contract and generate EMG signals. Different forces are applied to gripe three elastomer balls with the moduli of 0.21, 0.27 and 0.33 GPa, respectively. The corresponding gripping forces imposed on the balls were measured using a commercial optoforce sensor (Optoforce 3-axis force sensor) (Supplementary Fig. S20).

The peak-to-peak amplitude and the signal intensity are consistent with the gripping force (Fig. 6b, c). The EMG signal using the PWS electrodes is comparable to that with the Ag/AgCl gel electrode (Supplementary Fig. S21). The detection of EMG signals for muscle motion can have essential applications in the human–machine interfaces. For example, the EMG signal of a hand opening/closing from the PWS electrodes can serve as a user interface to control the opening and closing of an anthropomorphic robotic hand in a real-time manner^58^ (Fig. 6d, Supplementary Movie 7). Apart from the significant motion of a bicipital muscle, the PWS electrode can also detect the low-amplitude EMG signal produced by a finger performing flexion or extension (Fig. 6e, f).

**Fig. 7:**
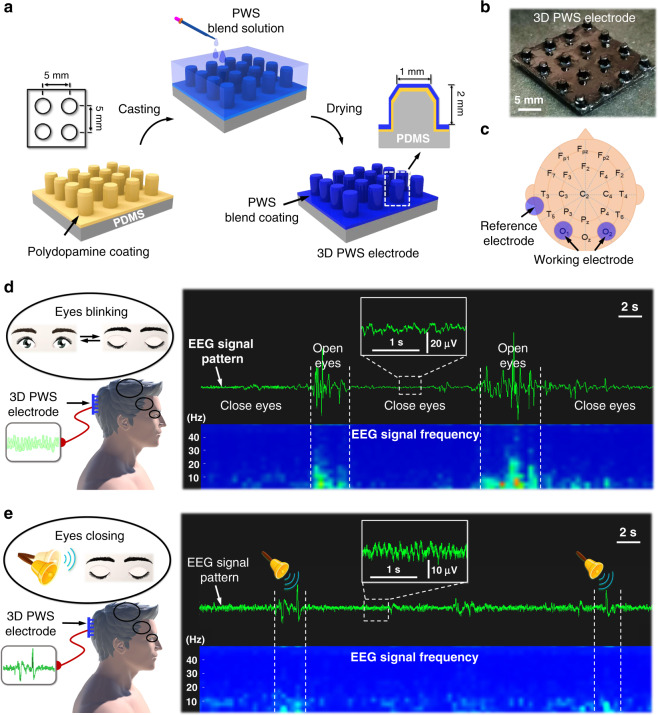
3D PWS electrodes with micro-pillar structures for EEG detection. **a** Fabrication of the 3D PWS electrodes. **b** Photo of a 3D PWS electrode. **c** Positioning two 3D PWS electrodes at the O1 and O2 sites of the rear head and a PWS film electrode behind the ear as the reference electrode. **d** EEG signals collected during eye-blinking. **e** EEG signals respond to auditory stimuli.

Compared with ECG and EMG, recording high-quality EEG signals is much more challenging due to the weak signal strength in the microvolts range, interference of scalp, and dense hair. In order to achieve decent contact with the hairy scalp, a 3D PWS dry electrode with vertical pillars was fabricated (Fig. 7a and Supplementary Fig. S22). These vertical pillars with a height of 2 mm and a diameter of 1 mm were arranged in a square array with an inter-pillar spacing of 5 mm (Fig. 7b). The pillars do not enhance the adhesiveness but can penetrate through the dense hair to contact the scalp.

To collect the EEG signals at the occipital lobe, two 3D PWS electrodes were mounted at the O1 and O2 sites of the rear head according to the 10–20 system (EEG)^57^, and another PWS film electrode was placed behind the ear as a reference electrode (Fig. 7c). To avoid auditory interferences, the volunteer sat in a comfortable position and relaxed by listening to the white noise. The potentials triggered by the optic nerves during the opening and closing of the eyes were detected. The biopotential falls in the frequency ranges of 7–15 Hz during the eye closure, the characteristic alpha waves (Fig. 7d). In contrast, when the eyes are opened, the EEG signals have a broader frequency range. The EEG waves are sensitive to the external sound stimuli. To capture the auditory response, the volunteer sat in a comfortable position and blindfolded to avoid visual interferences. While the eyes were closed, a loud bell was rung at random intervals, and the perturbed EEG signal with different frequency range was captured as responding to the auditory stimuli (Fig. 7e)^52,57^.

### Clinical setting study

**Fig. 8:**
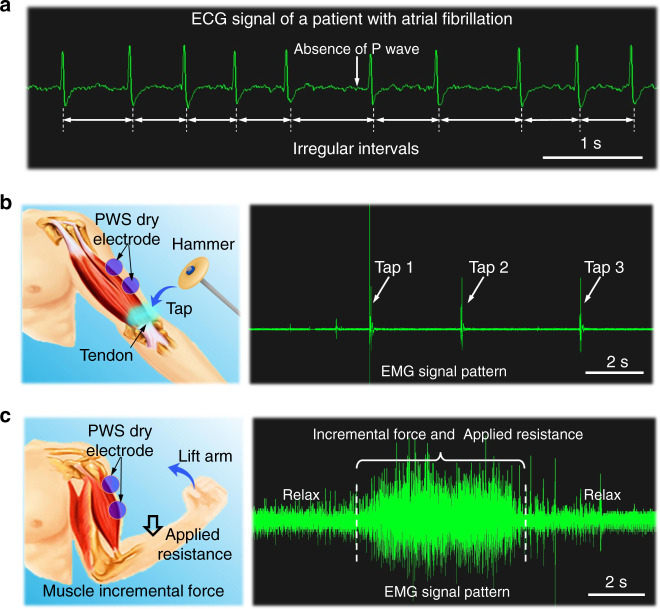
Clinical evaluation of PWS electrodes for ECG and EMG. **a** ECG signals showing the variability in the R-R intervals and absent P-waves, which are diagnostic of atrial fibrillation. **b** EMG signals showing a brief and significant increase in muscle potentials detected using the PSW dry electrode by tapping on the biceps tendon. **c** EMG signals showing incremental potentials during contraction of the biceps muscles, which decreased following relaxation.

The PWS dry electrodes were further mounted on a patient with atrial fibrillation in a clinic setting to examine the ability of the PWS dry electrodes in identifying electrocardiographic arrhythmia, detecting brief but significant increases in muscle activity during deep tendon reflex testing, and detecting sustained muscle activity during contraction against resistance and during relaxation. The ECG pattern distinctly indicates the absence of typical P peaks and irregular R-R interval (Fig. 8a, Supplementary Movie 8), consistent with the symptom of atrial fibrillation.

In addition, the EMG signals can be used to diagnose the muscle functions of neurological patients. Two PWS dry electrodes were mounted to a patient’s upper arm with a separation of 10 cm. When the biceps were triggered by tapping on the biceps tendon, the PWS dry electrodes sensitively recorded an immediate increase in muscle activity induced by contraction (Fig. 8b, Supplementary Movie 9). In another clinical testing, the patient tried to lift his forearm (under the pulling of biceps contraction) while an incremental external force was applied continuously on his forearm. In this case, an increase in muscle activity persisted during the continuous contraction of the biceps. The PWS dry electrode mounted on the biceps can accurately detect the increment of the EMG signal during continuous contraction and the signal decline in the following relaxation (Fig. 8c). These results suggest the ability of the PWS electrodes to quantify muscular strength for neurological assessments in clinical.

### Advantages of the PWS dry electrodes

The PWS electrodes have high conductivity, high mechanical stretchability, excellent adhesiveness to skin and excellent biocompatibility. They are different from other dry electrodes in literature. Nanocomposites with conductive nanofillers in the elastomer matrix can have high stretchability and high conductivity^41^, and they have been studied as dry electrodes for epidermal biopotential measurement^13,16,59,60^. However, the nanocomposite dry electrodes usually give rise to much higher electrode-skin impedance than the PWS electrode because the conductive nanofillers are the minority in the nanocomposites and their effective contact area to skin is thus actually very small^43^. In addition, they are usually not adhesive, and thus high motion artifacts can be observed. Another concern is the possible toxicity of the nanofillers.

The PWS blends are also different from the stretchable PEDOT:PSS composites reported in the literature. Stretchable PEDOT:PSS composites were obtained by adding additives^26,61–63^. For example, Bao et al. found that ionic liquids can significantly increase the stretchability and conductivity of PEDOT:PSS^26^. However, the stretchable PEDOT:PSS composites are not adhesive. They can give rise to high motion artifacts due to the poor skin-electrode contact during body movement^52,64,65^. In addition, additives like ionic liquids^66^ are toxic, so that PEDOT:PSS added with ionic liquids cannot be used for epidermal biopotential measurement. Although other stretchable PEDOT:PSS composites were used as the dry electrodes, they are not adhesive and thus give rise to high noise during body movement. Some soft adhesive electrodes were reported in literature^59,67^. For instance, ultrathin electrodes can be adhesive to skin. But they are difficult to handle, and high noise was observed during body movement. Apart from dry electrodes, conductive hydrogels were investigated as adhesive electrodes as well^68^. Because they are wet electrodes, the water vaporization from the hydrogels can induce signal decay and noise. They are not suitable for long-term use as well.

## Discussion

Herein, the blends film of PEDOT:PSS, WPU and D-sorbitol is prepared by solution processing. The resulted PWS films have high conductivity, self-adhesiveness, mechanical flexibility/stretchability and biocompatibility. The PWS film electrodes possess low skin-electrode interfacial impedance and excellent skin-compliance. They can be thus used to acquire high-quality epidermal biopotential signals, including ECG, EMG, and EEG, under various skin conditions. Moreover, the biopotential signals can be immune to motion artifacts. The PWS dry electrodes exhibit remarkably lower skin-electrode impedance and higher signal quality than other dry electrodes in literature. To collect high-quality EEG signals, PWS electrodes with micro-pillar structures were fabricated to establish secure contact with the scalp through dense hair. The EMG signal using dry electrodes can be used to control the motion of an anthropomorphic robotic hand. To further explore potential applications of these dry electrodes, a clinical study was performed in a patient with atrial fibrillation to identify electrocardiographic arrhythmia, brief but significant increase in muscle contraction during tendon hyper-flexion testing and sustained the increase in muscle contraction against resistance before normalizing during relaxation. The PWS dry electrodes display high adaptability to various conditions and precisely record the epidermal biopotential signals. They have advantages over the commercial Ag/AgCl electrode and other dry electrodes in literature. Therefore, they can be used for long-term healthcare monitoring of patients with regular daily life, rehabilitation, and humanoid robotic instruments.

## Methods

### Materials

WPU aqueous dispersion (WPU-3-505G) was supplied by Taiwan PU Corporation. The WPU (WPU-3-505G, 39.8 wt%) is a nonionic polyurethane and is used to prepare adhesive blend film with PEDOT and D-sorbitol. PEDOT:PSS aqueous solution (Clevios PH 1000 Lot 2015P0052) was purchased from Heraeus Co. The concentration of PEDOT:PSS was 1.3 wt% in the solution, and the weight ratio of PSS to PEDOT is about 2.5:1. D-sorbitol (97%) and ethylene glycol were obtained from Sigma-Aldrich. Polydimethylsiloxane (PDMS, Sylgard184) and curing agents were obtained from Dow Corning Company. All the chemicals were used as received without further purification.

### Preparation of PWS films

The PEDOT:PSS solution was mixed with a D-sorbitol aqueous solution and stirred for 30 min at room temperature. Subsequently, ethylene glycol and WPU solution (10 wt%) was added and further stirred for 1 h at room temperature. The PWS films were prepared by drop-casting the above blend solution into a petri dish and dried at 60 ^o^C for at least 2 h. Finally, the resultant PWS films were peeled off after cooling down.

### Preparation of 3D PWS electrode for EEG measurement

A flat mold (3 cm × 3 cm) with a square array of cylindrical holes (1.5 mm diameter, 2 mm depth) was prepared using polylactic acid by virtue of a 3D printer (LulzBot’s TAZ 5 3D printer, Loveland, CO). The PDMS base agent blended uniformly with a curing agent at a weight ratio of 10:1 and cured in an oven at 70 ^o^C for one hour. After de-molding, a PDMS substrate with pillar structures was obtained. In order to achieve a wettable surface for the PWS blend solution, a layer of polydopamine was coated on the PDMS substrate by immersing the substrate in the dopamine solution (pH 8.5) for 10 h. The resultant polydopamine-modified PDMS substrate was washed by deionized water and dropped with 4 mL of PWS blend solution consisting of PEDOT:PSS, WPU, and D-sorbitol. After drying at 60 ^o^C, a 3D PWS electrode with pillar structures was obtained for the EEG measurement.

### Materials characterization

The SEM images were collected using a Zeiss Supra 40 field emission scanning electron microscope. The AFM images were obtained using a Veeco NanoScope IV Multi-Mode AFM with the tapping mode. 3D optical microscopic observation was performed on a confocal laser scanning microscope (Carl Zeiss AG, LSM 700, Germany). The thickness of the polymer films was determined with an Alpha 500 step profiler. The impedance spectra were taken with an Autolab impedance analyzer with the dual-electrode method in the ranges of 1–10^4^ Hz. The two electrodes were placed on the forearm with a separation of 10 cm. The conductivities of the polymer films were measured with a four-point probe setup fitted with Keithley 2400 source/meter. In the conductivities shown in figures, the error bars represent the standard error.

### Mechanical characterization

The tensile measurements were conducted using an Instron Model 5500 Materials Testing System. The load cell is 100 N load cell, and the uniaxial strain was applied at a ramp rate of 1 mm/min. The load cell was calibrated prior to the testing.

### Adhesion force characterization

The adhesion force of a PWS film on the substrate was measured through the delamination process using a tensile testing machine (Instron Model 5500 Materials Testing System). A rectangle polymer film of 4 × 1 cm was laminated on the substrate. The polymer films were then delaminated perpendicularly to the substrate at a rate of 50 mm/min. The adhesion force was calculated in terms of the maximum stable force and the polymer film width. In the plots of the adhesion force, the error bars represent the standard error.

### Biopotential signal extraction

The ECG signals were acquired by placing two PWS film electrodes on the inner wrists and a reference electrode on the rear hand. The electrodes were connected to a signal-recording setup processed with a bandpass filter (0.5–150 Hz). The ECG signals were analyzed using the Matlab envelope function. The EMG tests were conducted by mounting two PWS electrodes on the upper arm or forearms and a PWS film as a reference electrode on the rear hand for the signal generated by bicipital or brachioradialis muscle, respectively. For the EMG signals collected for finger flexion and extensions, two PWS electrodes were placed on the forearms. In the EEG measurements, the PWS electrodes with pillars were placed at the O1 and O2 sites according to the 10–20 system of electrode placement on the head. Another PWS film was put on the back of the ear as the reference electrode.

There are two parts to the signal recording setup, including a microcontroller (Arduino UNO microcontroller) and a detector (Muscle SpikerBox Pro). Through potential differences between the working electrodes on the object area and the reference electrodes, the biopotential signals (ECG, EMG, and EEG) are captured by the Spikershield box. The signal processing algorithms are performed on the collected data using Matlab for fundamental signal analysis (Root-Mean-Square/Spectrogram/Fast-Fourier Transform).

Motion artifact measurement of PWS dry electrode during ECG signal recording. A coin button-type cellphone micro vibrator motor with a 1.1 cm^2^ area is used to generate analogous skin shaking. The vibrator (OEM, JMM181-BY1234BZ3V26L) provided by Yichang Baoyuan Electronics CO. LTD, China) works at a direct voltage of 3 V (/0.1 A), and the rated speed is about 12,000 ± 2500 rpm. The incident skin oscillation amplitude is about 1.5 mm. The vibrator is attached to the inner side of the forearm while the PWS dry electrodes are fixed on the inner wrist. The ECG signal is recorded regularly when the distance between vibrator and PWS electrode is changed to 5, 3, and 1 cm, respectively. The RMS analysis of the ECG signal is performed for evaluating the signal noise and resistance against motion artifact.

## Supplementary information

Supplementary Information

Description of Additional Supplementary Files

Supplementary Movie 1

Supplementary Movie 2

Supplementary Movie 3

Supplementary Movie 4

Supplementary Movie 5

Supplementary Movie 6

Supplementary Movie 7

Supplementary Movie 8

Supplementary Movie 9

## Data Availability

The data that support the findings of this study are available in the main text and the Supplementary Information. The source data for Figs. 2h, i, 3a, b, c, 4f, h, 5c, f, and Supplementary Figs. S8, S9, S10a, S11a, S11c, S12, S13, S14, S16 and S17a are provided as a Source Data file.

## Code availability

The codes for ECG signal recording and analysis used in this study are available from the corresponding authors upon request.
