# Supplementary material for: Fully organic compliant dry electrodes self-adhesive to skin for long-term motion-robust epidermal biopotential monitoring
Source: Nat Commun. 2020 Sep 17;11:4683. doi: 10.1038/s41467-020-18503-8 (PMC7499260; doi:10.1038/s41467-020-18503-8)
Supplement: Supplementary file 1 — Supplementary Information [file 41467_2020_18503_MOESM1_ESM.docx]

**Supplementary Information**

Fully Organic Compliant Dry Electrodes Self-adhesive to Skin for Long-term Motion-robust Epidermal Biopotential Monitoring

Zhang et al

Supplementary Figure 1-22

Supplementary Table 1

**Supplementary Figures**


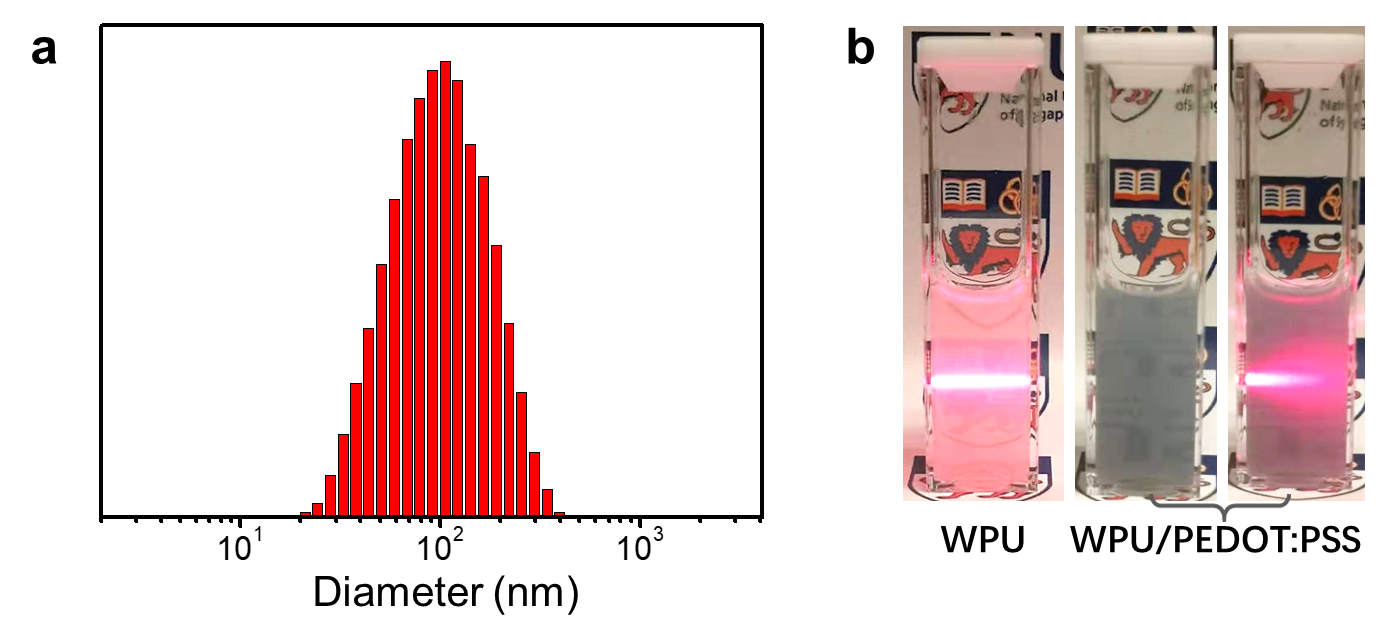


**Supplementary Figure 1**. **Solutions of WPU, PEDOT:PSS and their mixture**. **a** Dynamic light scattering analysis of the WPU solution (2 wt%); **b** Photos of the WPU solution, PEDOT:PSS solution and their mixture.


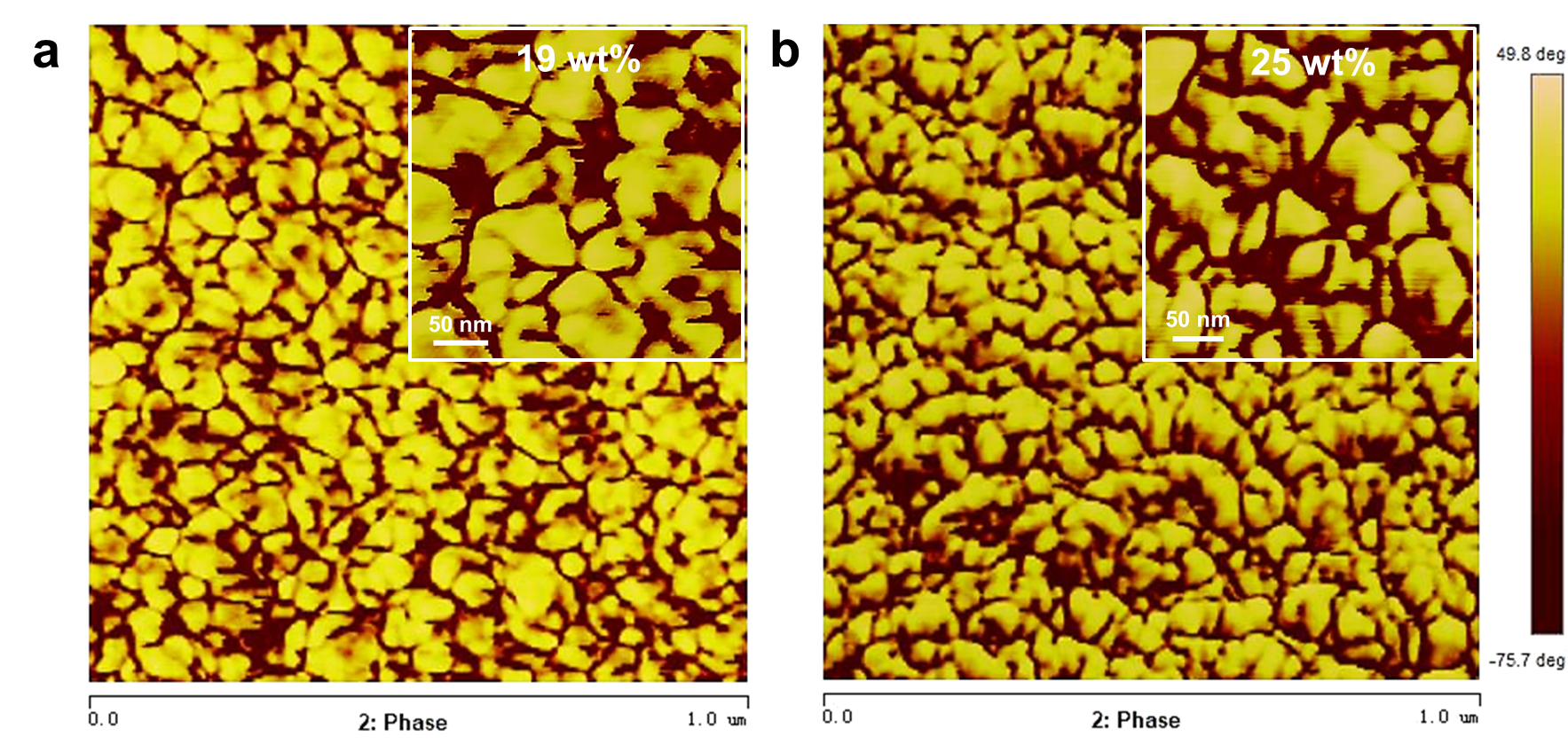


**Supplementary Figure 2**. **Phase AFM images of a PWS film**. **a**, **b** AFM phase images of PWS film with PEDOT:PSS loading of 19 wt% and 25 wt%, respectively.


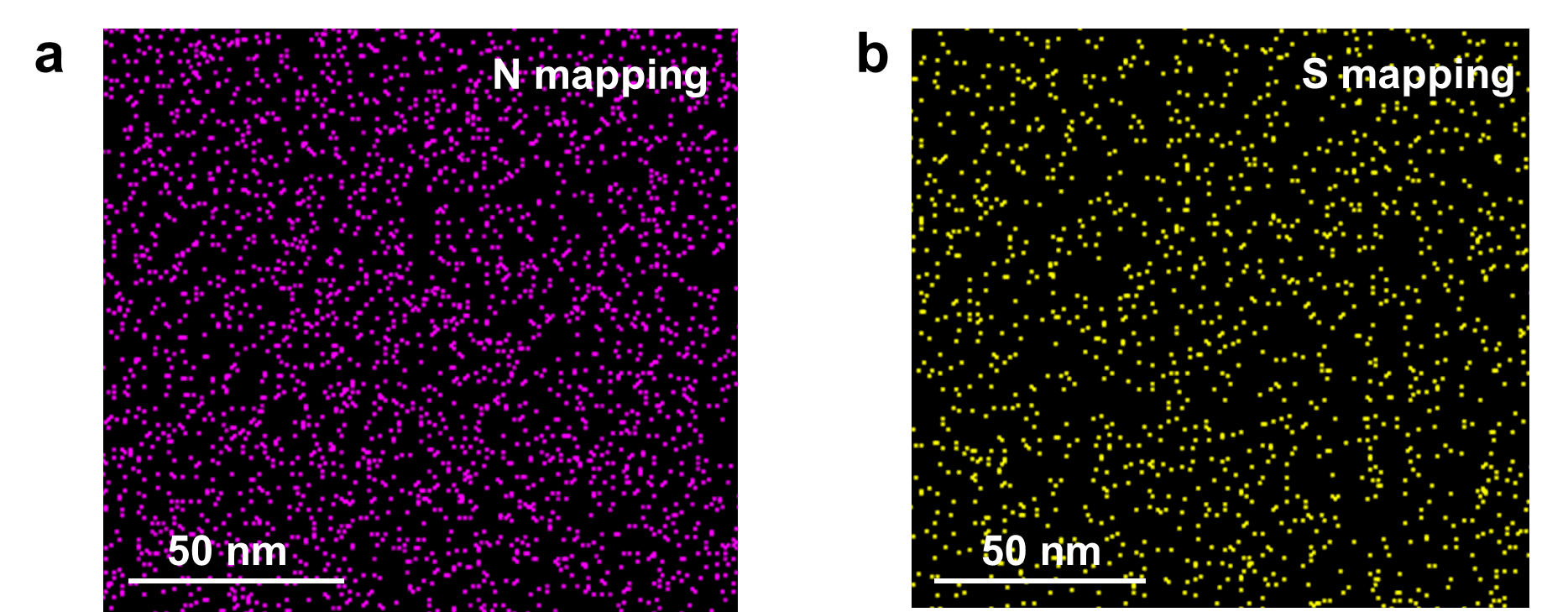


**Supplementary Figure 3**. **Energy-dispersive X-ray (EDX) analysis of a PWS film. a**, **b** EDX images of nitrogen (N) and sulfur (S) in the surface of PWS blend film with PEDOT:PSS loading 19 wt%.


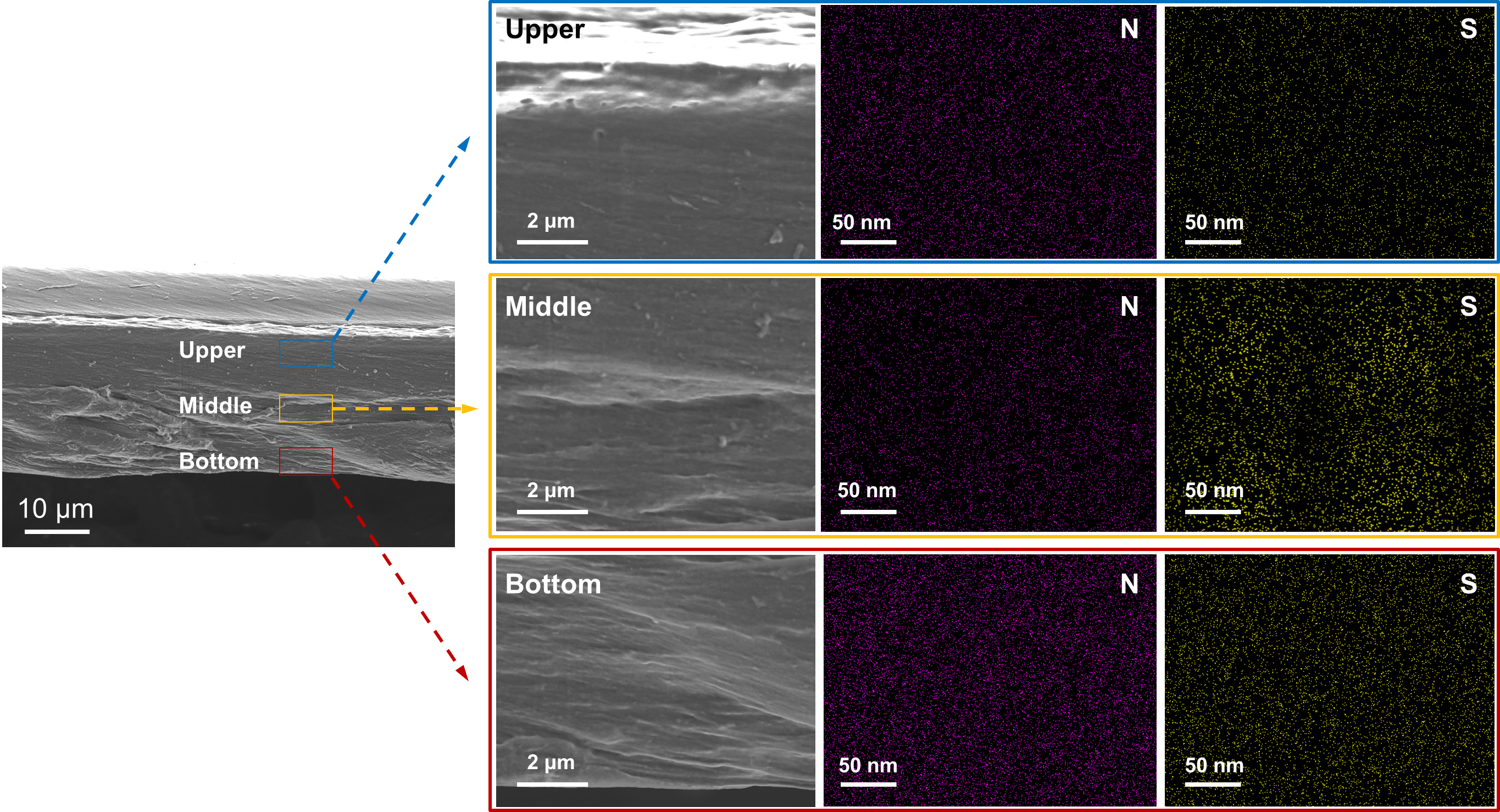


**Supplementary Figure 4**. **Cross-section** **SEM images and EDX analysis of a PWS film.** The SEM images and N, S elements mapping in the upper, middle, and bottom area in the cross-section of the blend film with a loading of 19 wt% PEDOT.


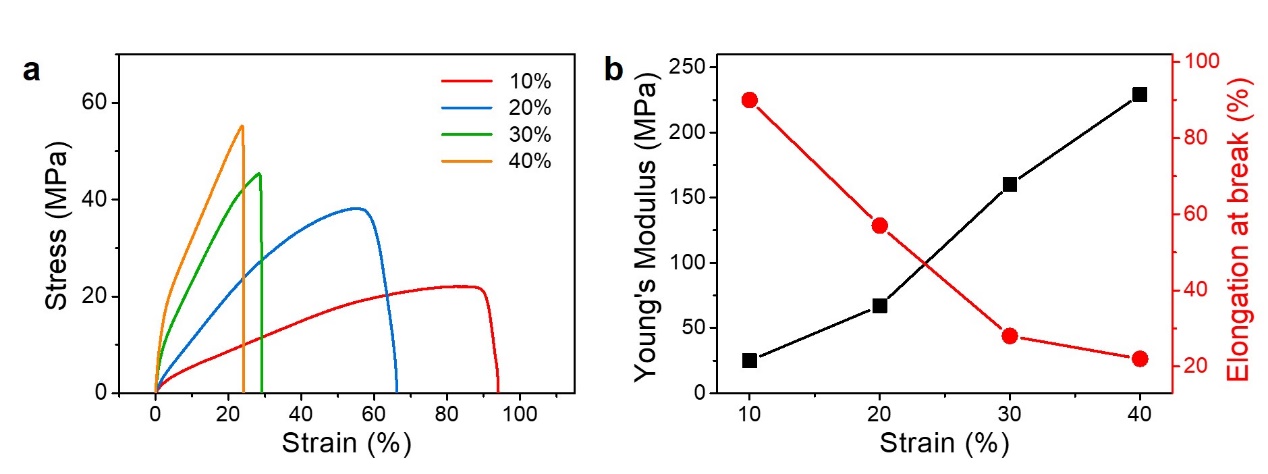


**Supplementary Figure 5**. **Mechanical properties of PEDOT:PSS/WPU (PW) films.** **a** Stress-strain curves of PW films without D-sorbitol and the PEDOT:PSS loadings of 10 wt%, 20 wt%, 30 wt% and 40 wt%. **b** Variations of Young’s modulus and elongation at break of PW films without the addition of D-sorbitol in the films.


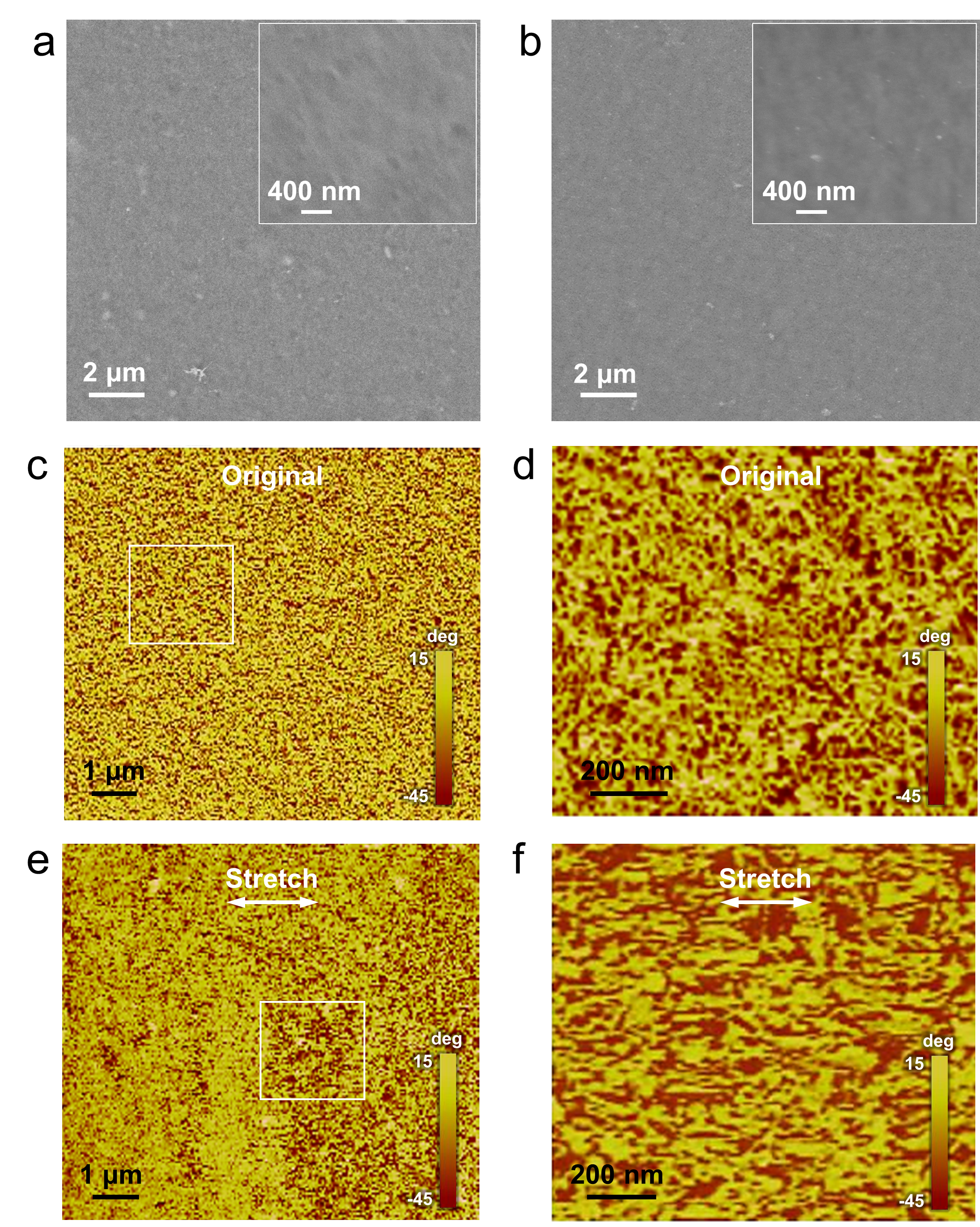


**Supplementary Figure 6**. **SEM image and phase AFM image of a stretched PWS film**. SEM image of a PWS film (a) un-stretched and (b) stretched at a 30 % strain. **c, d** The phase AFM image of a pristine PWS blend film. **e**, **f** The phase AFM image of a stretched PWS film at a 30% strain. d and f are magnifications of the areas marked in c and e, respectively.


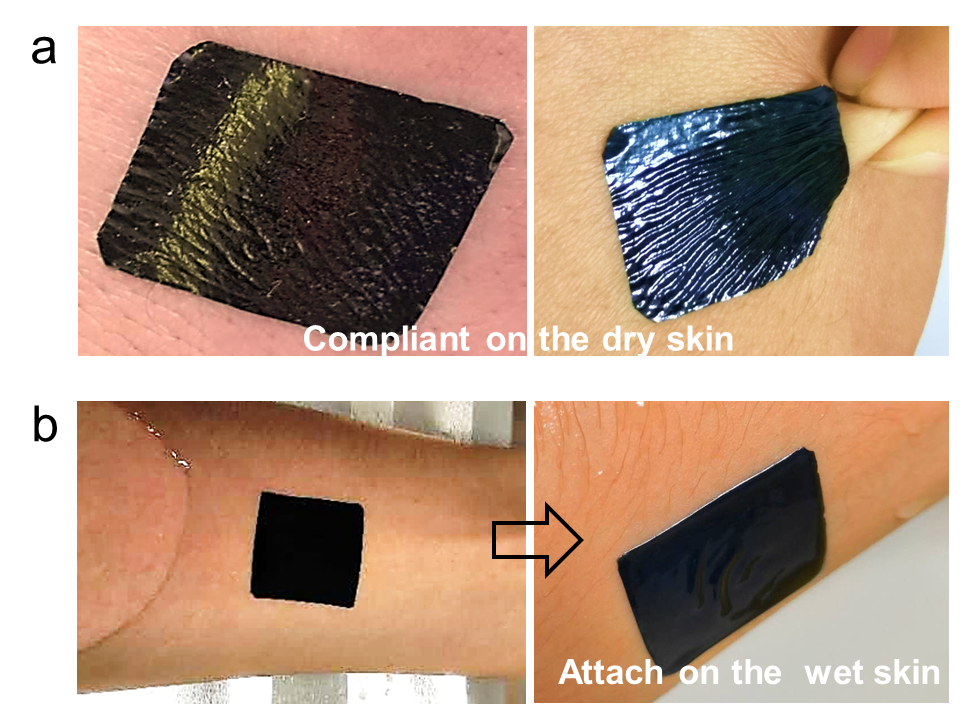


**Supplementary Figure 7**. Photos to indicate the adhesiveness of PWS films on the dry and wet skin.


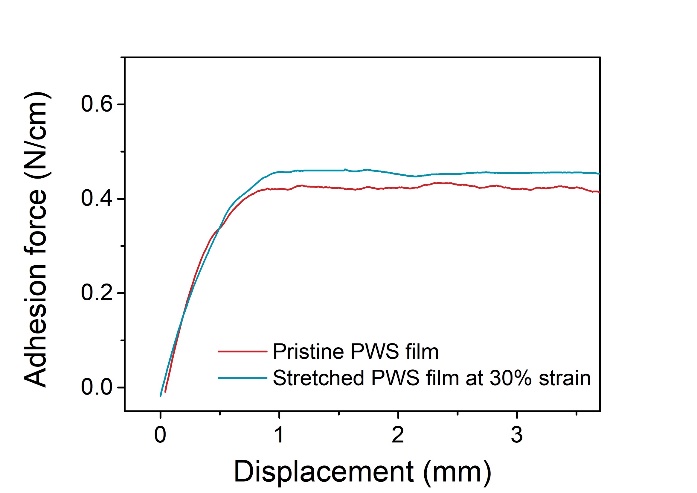


**Supplementary Figure 8**. **Strain effect on the adhesion**. The adhesion forces of a PWS film un-stretched and stretched at a strain of 30% to skin.


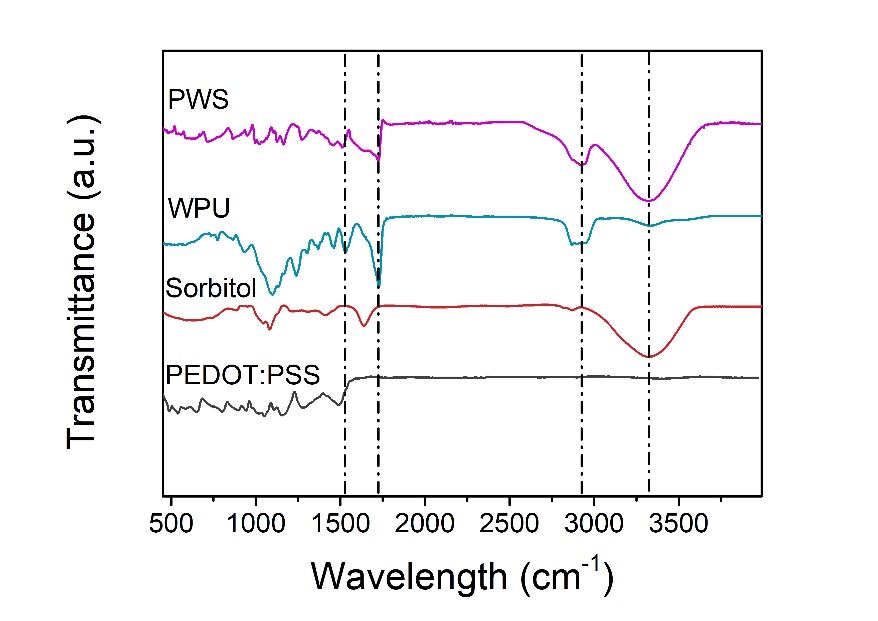


**Supplementary Figure 9**. IR reflectance spectra of PEDOT:PSS, D-sorbitol, WPU and a PWS film.


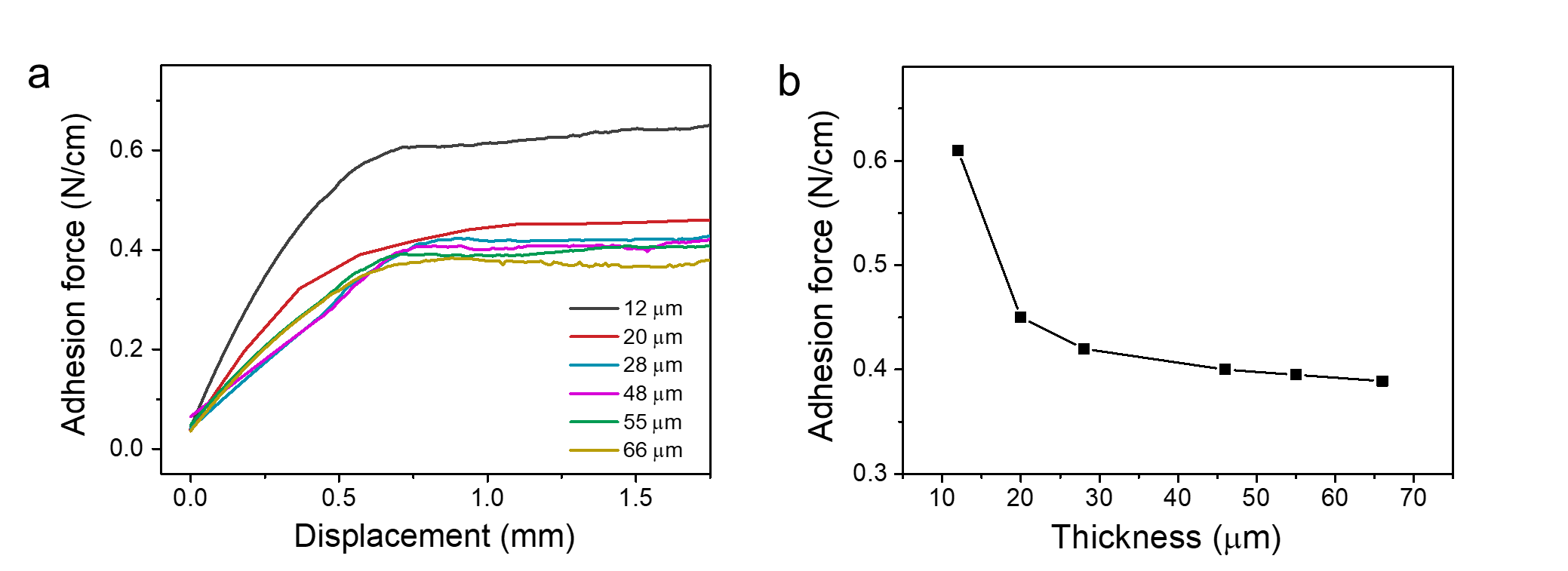


**Supplementary Figure 10**. **a**, **b** Adhesion forces of PWS films of different thicknesses to skin.


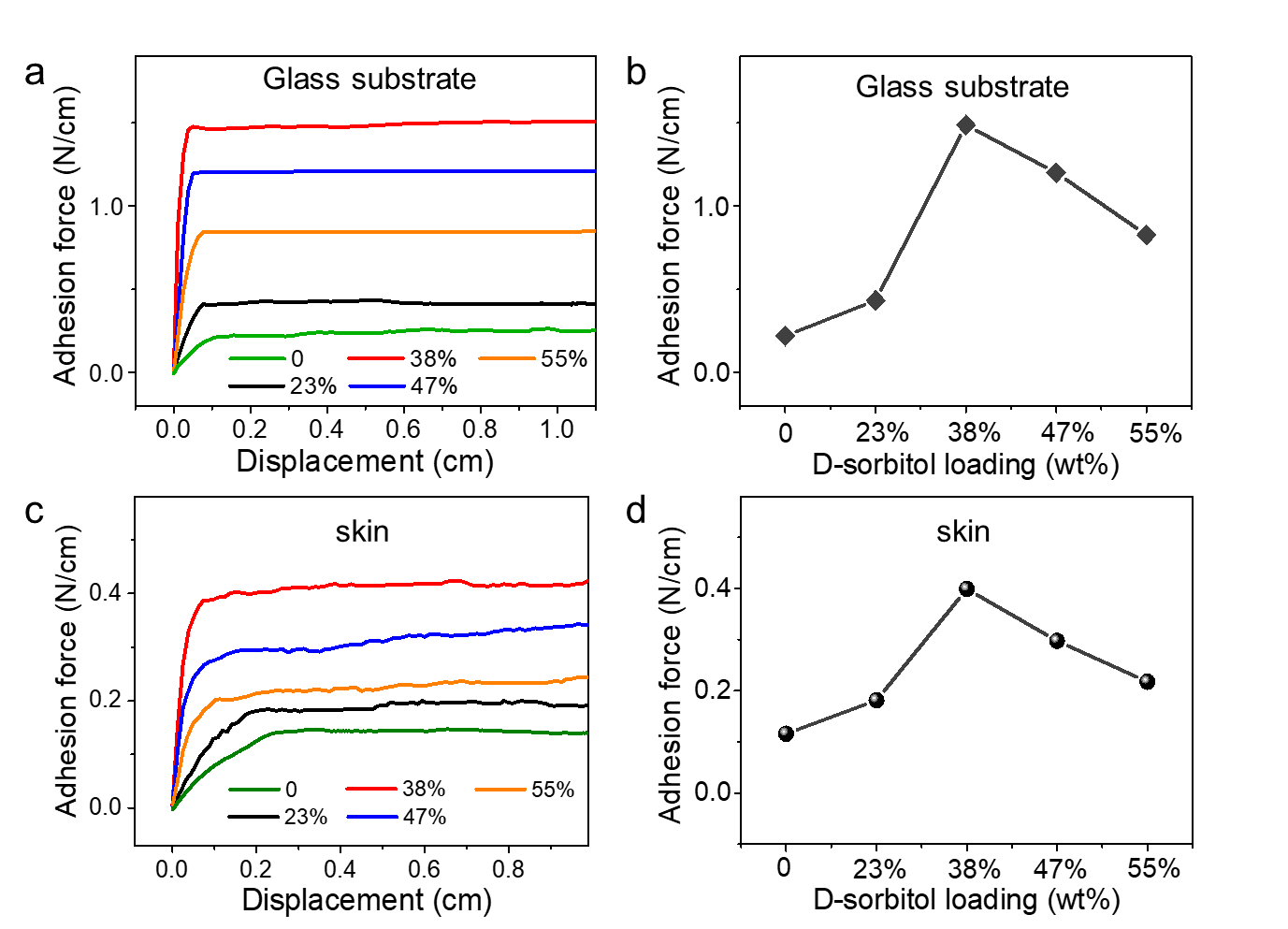


**Supplementary Figure 11**. **Adhesion force of PWS films with different D-sorbitol loadings**. **a**, **c** Adhesion forces of PWS films with different contents of D-sorbitol when delaminated from the surface of the glass and skin, respectively. **b**, **d** The maximum adhesion force of PEDOT film on the glass surface and skin, respectively.


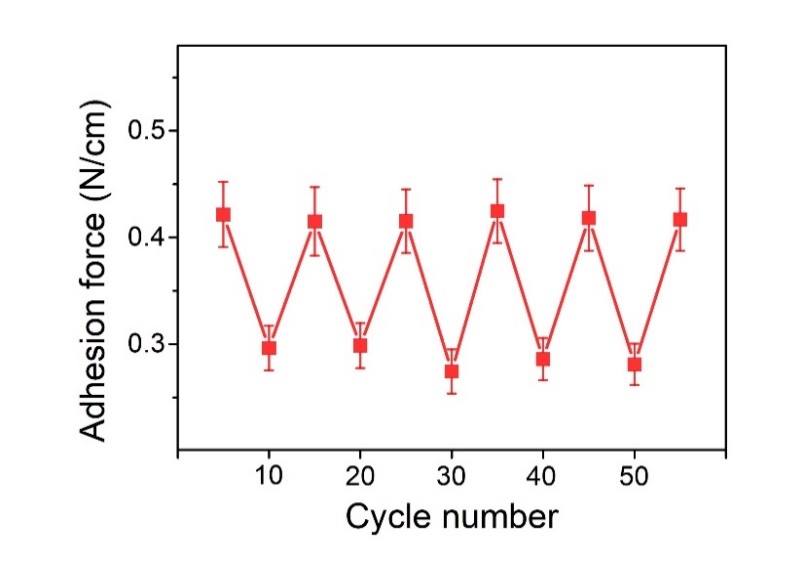


**Supplementary Figure 12**. Adhesion force variation of PWS film on the skin for 50 attach/detach cycles.


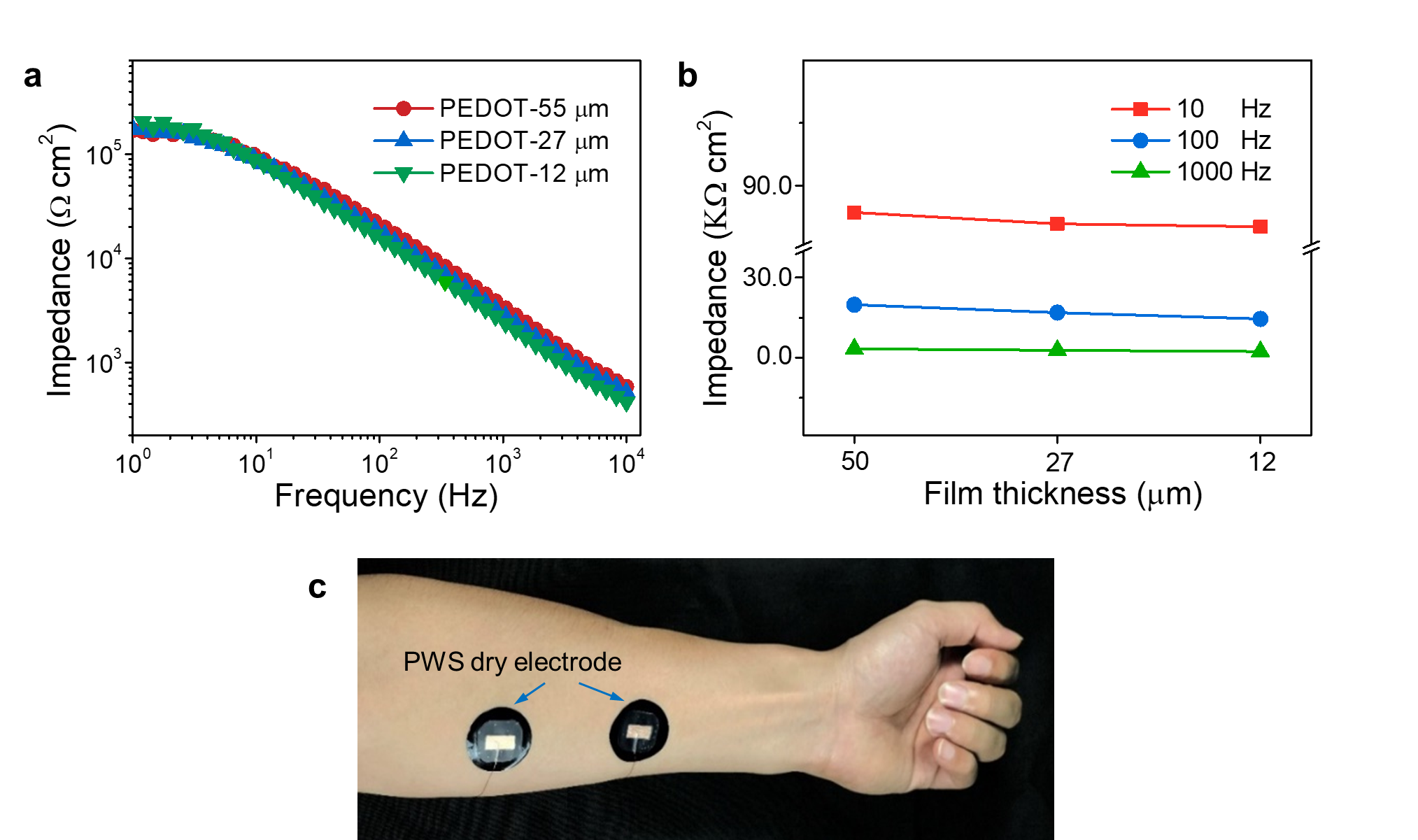


**Supplementary Figure 13**. **Impedance spectra of PWS dry electrode on the skin**. **a** Impedance spectra of PWS blend films with different thicknesses on skin. **b** Impedances at 10, 100 and 1000 Hz. **c** Placement of two PWS electrodes on the skin for the impedance measruements.


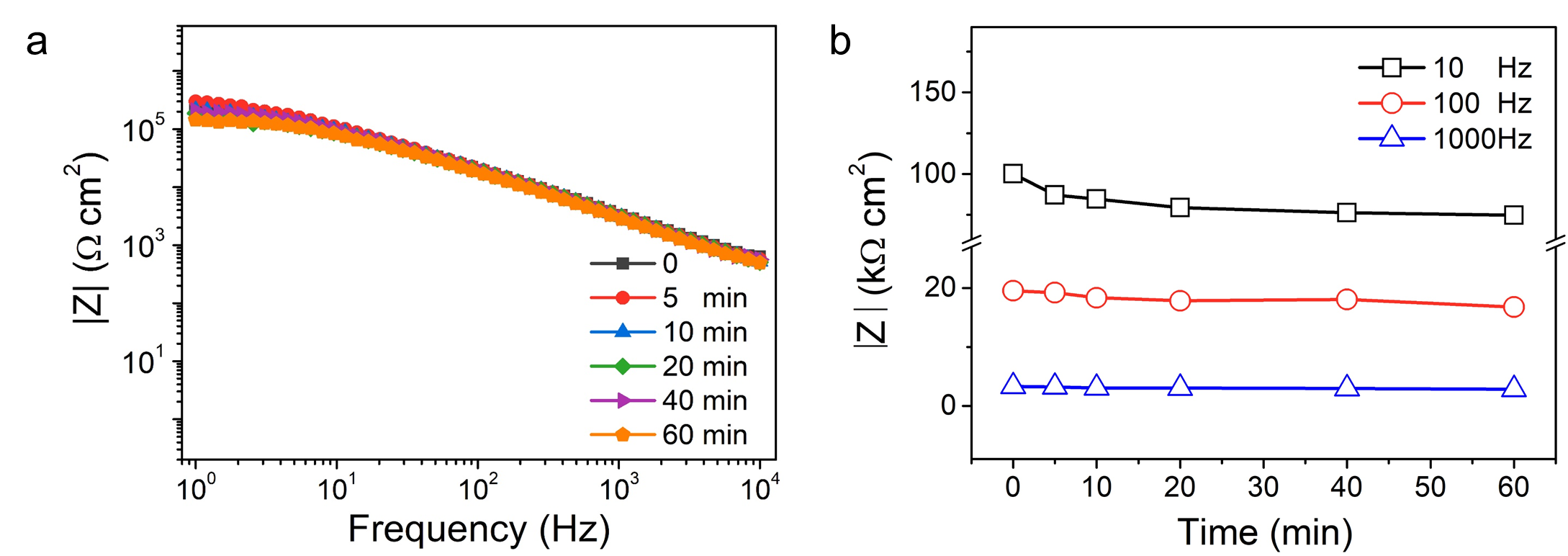


**Supplementary Figure 14**. **a**, **b** Impedance variation of PWS dry electrode attached to the skin within 1 hour.


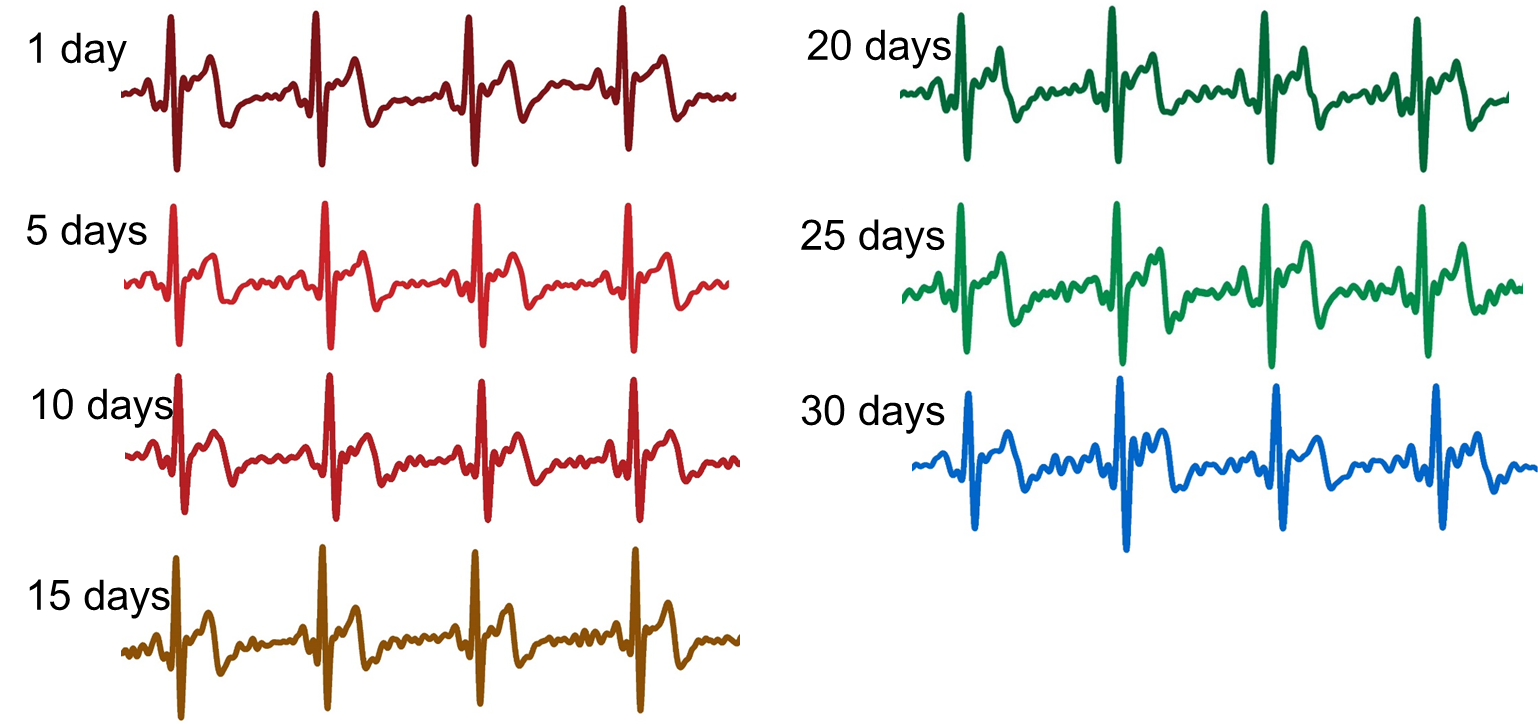


**Supplementary Figure 15**. **Long-term ECG detection using PWS electrodes for 30 days**. Two PWS electrode (3 cm diameter) were placed symmetrically on a volunteer’s inner wrists of the right and left arms, and another PWS electrode was attached on the back of the left hand as the ground electrode. The PWS electrodes are attached continuously on the skin for one hour and tested once every 20 min (three measurements for each test). The ECG signal is recorded one time each day under the same conditions. After the measurement, the PWS electrodes are peeled off carefully and cleaned by the medical alcohol swab. They are then stored in a vessel at ambient conditions.


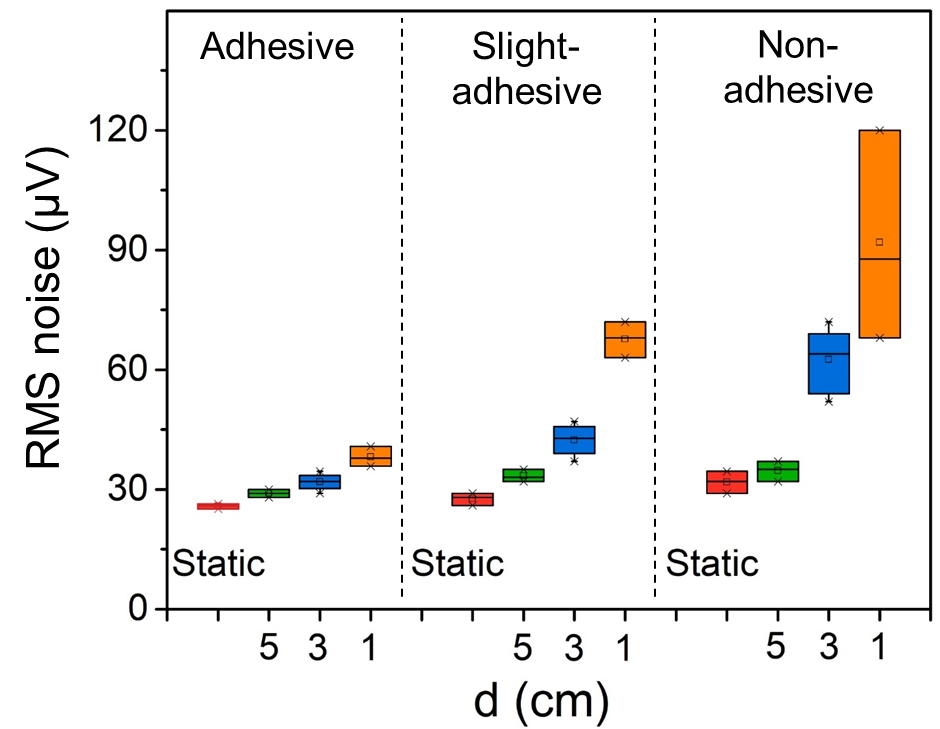


**Supplementary Figure 16**. **RMS noise analysis**. RMS noise produced by adhesive PWS electrodes, slight-adhesive PW electrode and non-adhesive PEDOT:PSS film electrode on the skin under motion induced by an electrical vibrator. The separation of the vibrator from the electrode is 5, 3, and 1 cm.


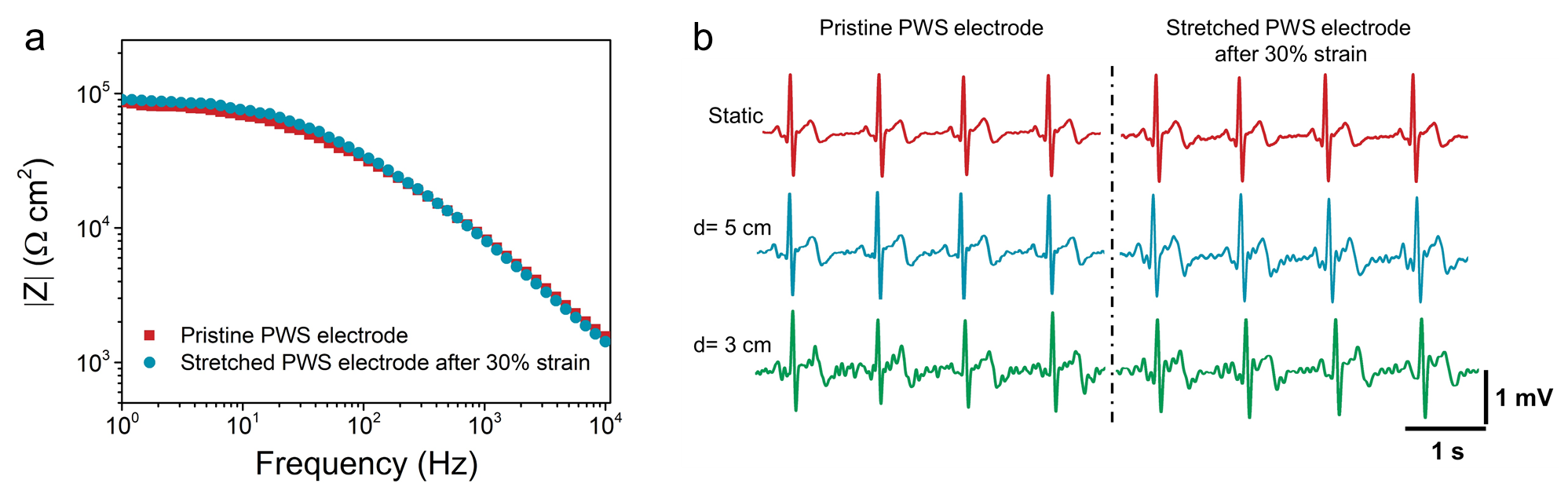


**Supplementary Figure 17**. **Impedance spectra and ECG using a stretched PWS electrode**. **a** The skin-contact impedance of a pristine PWS electrode and a stretched PWS electrode with a strain of 30%. **b** The ECG signals recorded using pristine PWS electrodes and PWS electrodes with hysteresis due to the stretching caused by an electrical vibrator. The separation of the vibrator from the electrode is 5 and 3 cm.


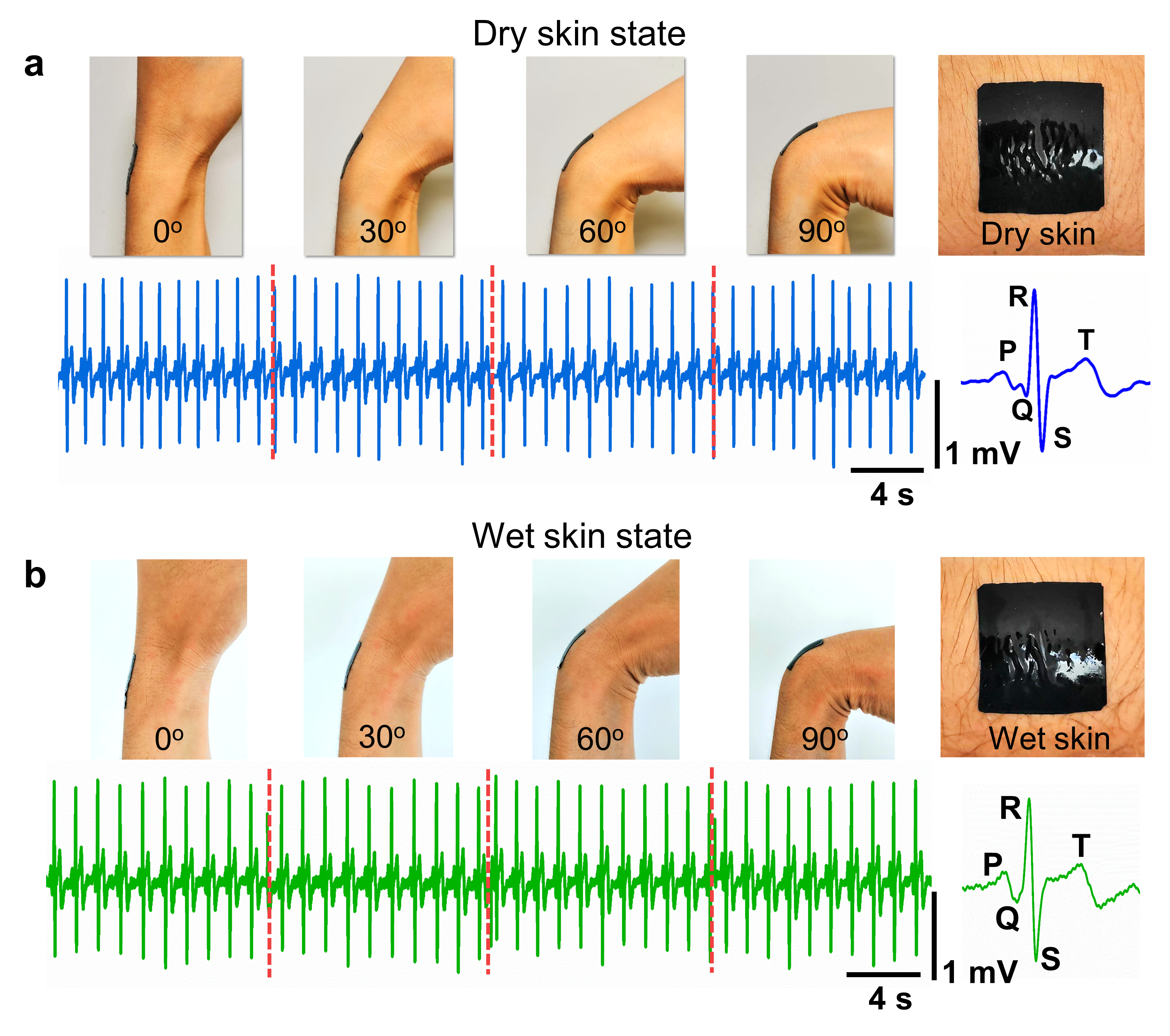


**Supplementary Figure 18**. **ECG measurements on dry/wet wrist**. **a**, **b** The ECG signals recorded by PWS dry electrodes on dry and wet skin during the wrist bending.


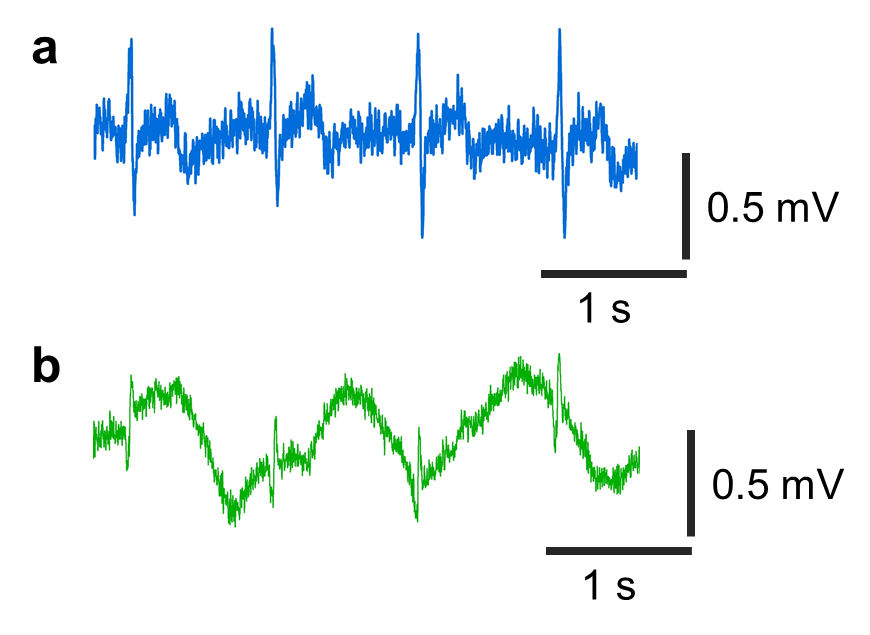


**Supplementary Figure 19**. **ECG detection in water**. **a** ECG signals using PWS dry electrode. **b** Commercial Ag/AgCl electrode in water. All the electrodes attached to the wrist and opisthenar, including the reference electrode, have emerged in water.


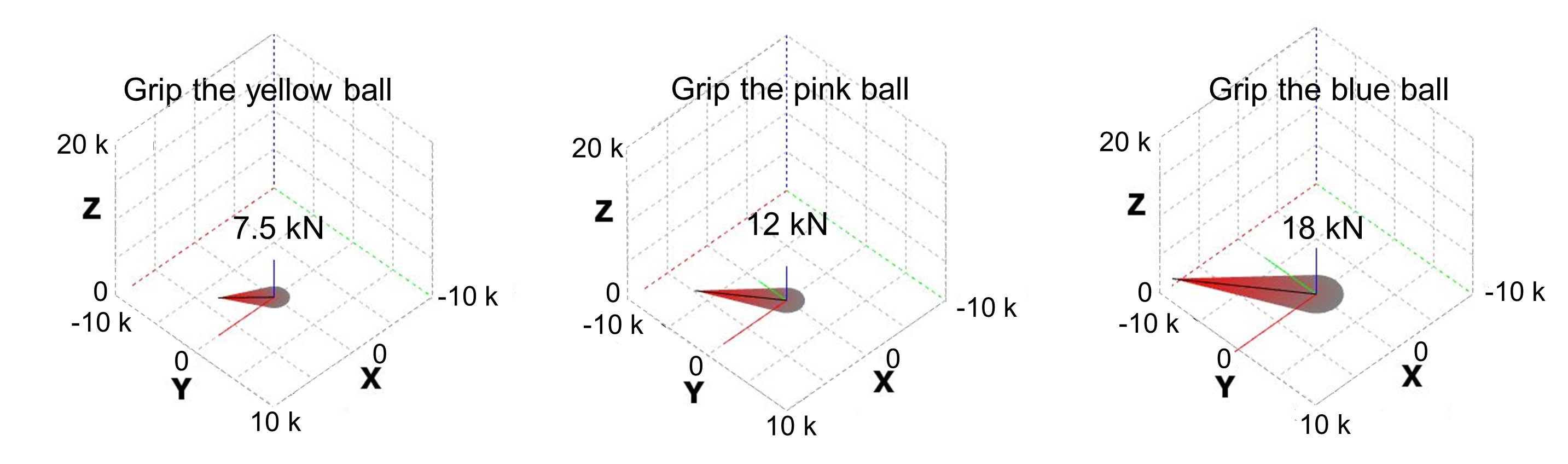


**Supplementary Figure 20**. **Gripping pressure force measurements**. Measurement on the gripping pressure force when gripped the yellow, pink, and blue elastic balls by a commercial optoforce sensor (Optoforce 3-axis force sensor).


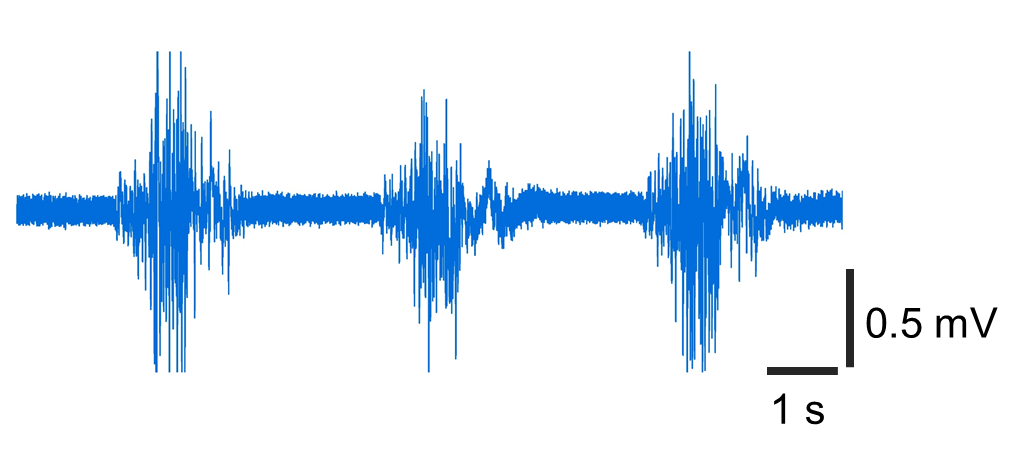


**Supplementary Figure 21**. **EEG signal recorded using commercial Ag/AgCl gel electrode**. EMG signal of the bicipital muscle of an upper arm during contraction and releasing by using commercial gel electrode.


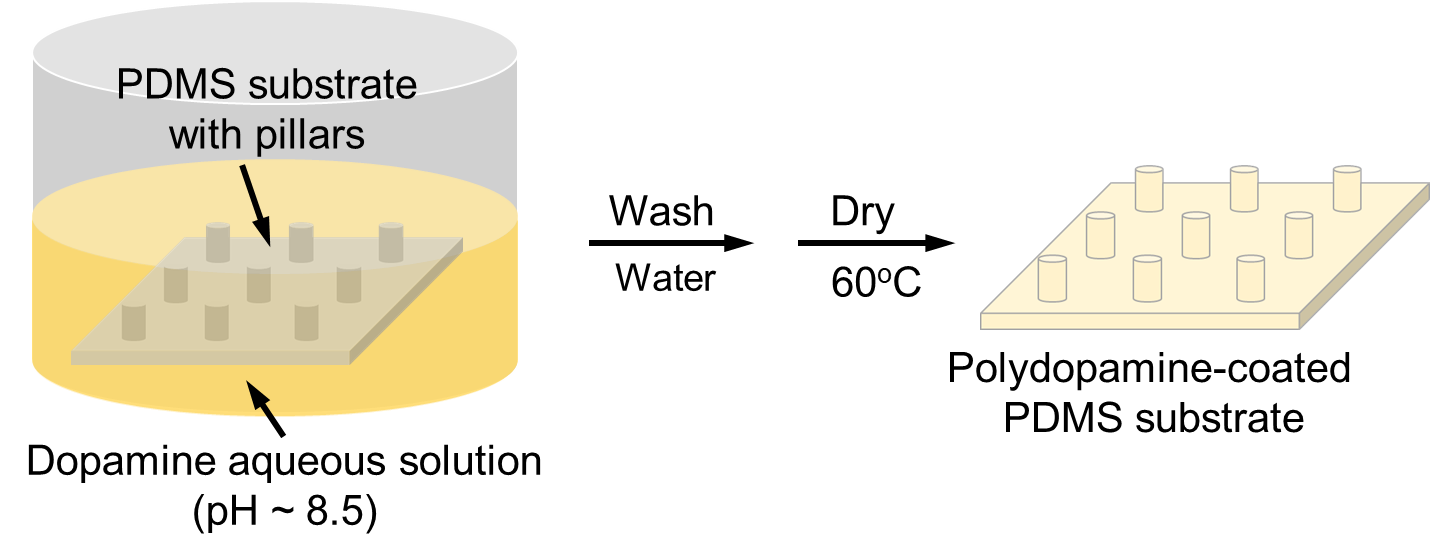


**Supplementary Figure 22**. **Polydopamine-modified substrate for EEG electrodes**. The surface of PDMS substrate with pillars is modified by polydopamine for the following coating with PEDOT:PSS/WPU/sorbitol blend solution.

**Supplementary Tables**

Supplementary Table 1. Stretchability, impedance, adhesion, and ECG detection performances of various dry electrodes.

| Electrodes | Stretchability | Impedance at 10Hz  (kΩ cm^2^) | ECG  amplitude  (mV) | Adhesion | Applicability | | | | | | Ref. |
| --- | --- | --- | --- | --- | --- | --- | --- | --- | --- | --- | --- |
|  |  |  |  |  | Static-state | | | Dynamic-state | | |  |
|  |  |  |  |  | Noise  (mV) | Baseline | | | Noise  (mV) | Baseline |  |
| PEDOT/  Polyimide | Not  applicable | ~200 | 0.60 | No | ~ 0.12 | | Normal | | Not applicable | | 1 |
| PEDOT:PSS  coated paper | Not  applicable | 400 | 0.80 | No | ~0.30 | | Wander | | Not applicable | | 2 |
| PEDOT film/  Ag/AgCl | 36 % | Not  applicable | 0.35 | No | ~ 0.08 | | Normal | | Not applicable | | 3 |
| PEDOT coated  textile | 10 % | 400 | 0.80 | No | ~ 0.40 | | Normal | | Not applicable | | 4 |
| PEDOT coated  textile | Low | 300 | 0.55 | No | ~ 0.05 | | Wander | | Not applicable | | 5 |
| Ag/PDMS with  cilia-patterns | Very low | Not  applicable | 0.76 | No | ~ 0.10 | | Normal | | Not applicable | | 6 |
| Ag nanowire/  PDMS/MPU blend | 50% | 150 | 0.4 | No | 0.05 | | Wander | | Not applicable | | 7 |
| Ag nanowire/  PDMS | 400% | 500 | 0.20 | Yes | ~ 0.05 | | Wander | | Not applicable | | 8 |
| Ag particles/  Ecoflex | Low | 105 | 0.25 | Yes | ~ 0.04 | | Normal | | 0.10 | Wander | 9 |
| Carbon nanofillers/ /PDMS with pillars | 100% | Not  applicable | 0.50 | Yes | ~ 0.20 | | Wander | | 0.10 | Wander | 10 |
| Carbon black/  PDMS with suckers | 100% | Not  applicable | 1.2 | Yes | ~ 0.20 | | Normal | | Not applicable | | 11 |
| Au thin  film/polyimide | Not  applicable | 500 | 1.77 | Yes | ~ 0.16 | | Normal | | Not applicable | | 12 |
| Au pattern/  Ecoflex/Silicon | 26% | 600 | 0.02 | Yes | 0.005 | | Normal | | Not applicable | | 13 |
| Au film/Parylene/  PU fiber | 20% | 30 | 1.4 | Yes | 0.052 | | Normal | | Not applicable | | 14 |
| Au pattern/Parylene | 30% | 250 | 0.3 | No | 0.079 | | Normal | | Not applicable | | 15 |
| AuGa/PDMS | 170% | > 200 | __ | Yes | __ | | __ | | Not applicable | | 16 |
| Self-adhesive PEDOT film | **43%** | **80** | **1.84** | **Yes** | **<0.025** | | **Normal** | | **<0.038** | **Normal** | **This**  **work** |

Supplementary References

1. Roberts, T. et al. Flexible inkjet-printed multielectrode arrays for neuromuscular cartography. *Adv. Healthcare Mater.* **5**, 1462-1470 (2016).

2. Bihar, E. et al. Inkjet-printed PEDOT:PSS electrodes on paper for electrocardiography. *Adv. Healthcare Mater.* **6**, 1601167 (2017).

3. Wang, K. et al. Stretchable dry electrodes with concentric ring geometry for enhancing spatial resolution in electrophysiology. *Adv. Healthcare Mater.* **6**, 1700552 (2017).

4. Pani, D. et al. Fully textile, PEDOT:PSS based electrodes for wearable ECG monitoring systems. *IEEE Trans. Biomed. Eng.* **63**, 540-549 (2016).

5. Sinha, S.K. et al. Screen-printed PEDOT:PSS electrodes on commercial finished textiles for electrocardiography. *ACS Appl. Mater. Interfaces* **9**, 37524-37528 (2017).

6. Jin, G.J., Uddin, M.J. & Shim, J.S. Biomimetic cilia-patterned pubber electrode using ultra conductive polydimethylsiloxane. *Adv. Funct. Mater.* **28**, 1804351 (2018).

7. Son, D. Bao, Z. N. et al. An integrated self-healable electronic skin system fabricated via dynamic reconstruction of a nanostructured conducting network. *Nat. Nanotechnol.* **13**, 1057-1065 (2018).

8. Kim, J.-H., Kim, S.-R., Kil, H.-J., Kim, Y.-C. & Park, J.-W. Highly conformable, transparent electrodes for epidermal electronics. *Nano Lett.* **18**, 4531-4540 (2018).

9. Stauffer, F. et al. Skin conformal polymer electrodes for clinical ECG and EEG recordings. *Adv. Healthcare Mater.* **7**, 1700994 (2018).

10. Kim, T., Park, J., Sohn, J., Cho, D. & Jeon, S. Bioinspired, highly stretchable, and conductive dry adhesives based on 1D–2D hybrid carbon nanocomposites for all-in-one ECG electrodes. *ACS Nano* **10**, 4770-4778 (2016).

11. Chun, S. et al. Conductive and stretchable adhesive electronics with miniaturized octopus-like suckers against dry/wet skin for biosignal monitoring. *Adv. Funct. Mater.* **28**, 1805224 (2018).

12. Nawrocki, R.A. et al. Self-adhesive and ultra-conformable, sub-300 nm dry thin-film electrodes for surface monitoring of biopotentials. *Adv. Funct. Mater.* **28**, 1803279 (2018).

13. Tian, L., Roger, J. R., et al. Large-area MRI-compatible epidermal electronic interfaces for prosthetic control and cognitive monitoring. *Nat Biomed Eng* **3**, 194-205 (2019).

14. Lee, S., Someya, T. et al. Ultrasoft electronics to monitor dynamically pulsing cardiomyocytes. *Nat. Nanotechnol.* **14**, 156-160 (2019).

15. Koo, J.H., Kim, D. H. et al. Wearable electrocardiogram monitor using carbon nanotube electronics and color-tunable organic light-emitting diodes. *ACS Nano* **11**, 10032-10041 (2017).

16. Dejace L., Laubeuf N., Lacour S. P., Gallium-based thin films for wearable human motion sensors. *Adv. Intell. Syst.* **1**, 1900079 (2019).
