# Supplementary material for: Fully organic compliant dry electrodes self-adhesive to skin for long-term motion-robust epidermal biopotential monitoring
Source: Nat Commun. 2020 Sep 17;11:4683. doi: 10.1038/s41467-020-18503-8 (PMC7499260; doi:10.1038/s41467-020-18503-8)
Supplement: Supplementary file 2 — Description of Additional Supplementary Files [file 41467_2020_18503_MOESM2_ESM.pdf]

Title: Supplementary Movie 1.
Description: Insensitivity of the PWS films to strains, shown by no change in intensity of the LED.

Title: Supplementary Movie 2.
Description: In the repeated stretching and releasing cycles, the PWS electrode exhibits small resistance variation with strain.

Title: Supplementary Movie 3.
Description: An adhesive PWS film bearing a weight of 250 g attaches tightly onto the ITO.

Title: Supplementary Movie 4.
Description: An adhesive PWS blend film is peeled off from the skin.

Title: Supplementary Movie 5.
Description: After repeated stretching/releasing cycles, the PWS films still keep stable adhesion on the glass plate and skin.

Title: Supplementary Movie 6.
Description: The PWS films attach tightly on a wrist that bends and twists vigorously and continuously.

Title: Supplementary Movie 7.
Description: The PWS dry electrode used to record the EMG signals and control the opening and closing of the anthropomorphic robotic hand remotely.

Title: Supplementary Movie 8.
Description: ECG waveform of a patient with clinically diagnosed Atrial Fibrillation.

Title: Supplementary Movie 9.
Description: EMG signals obtained from the patient during the Elbow jerk response.
